# Research Progress of Ionic Liquids Hybridized with Porous Materials for CO_2_ Capture: From Bulk to Confinement-Enhanced Adsorbents

**DOI:** 10.3390/nano16120727

**Published:** 2026-06-11

**Authors:** Enqi Zhang, Zhenzhen Wang, Yanwei Chi, Zhiyong Li

**Affiliations:** Henan Key Laboratory of Green Chemistry, Collaborative Innovation Center of Henan Province for Green Manufacturing of Fine Chemicals, Key Laboratory of Green Chemical Media and Reactions, Ministry of Education, School of Chemistry and Chemical Engineering, Henan Normal University, Xinxiang 453007, China; zhangeqi@stu.htu.edu.cn (E.Z.); wangzhenzhen@htu.edu.cn (Z.W.)

**Keywords:** ionic liquids, carbon capture, porous materials, hybridization techniques, life-cycle assessment

## Abstract

The continuous rise in carbon emissions poses a serious threat to the global climate, driving the urgent need for efficient CCUS technologies. Ionic liquids (ILs), with their negligible vapor pressure, excellent thermal stability, and tunable molecular structures, have emerged as promising materials for CO_2_ capture. However, the high viscosity of bulk ILs severely restricts gas mass transfer. To overcome this limitation, integrating ILs with porous materials featuring large surface areas and well-defined pore structures has emerged as a synergistic strategy, combining the high CO_2_ affinity and selectivity of ILs with the rapid mass transfer and structural stability of porous supports. This review systematically summarizes the CO_2_ capture mechanisms and limitations of bulk ILs and further highlights recent advances in the design, synthesis, and applications of IL-based hybrid adsorbents. Particular attention is given to confinement-enhanced mechanisms, whereby nanoscale confinement fundamentally alters the physicochemical behavior of ILs, transforming them from disordered bulk liquids into ordered, interface-dominated systems. In addition, the life-cycle assessment and techno-economic analysis of IL hybrid systems are critically evaluated.

## 1. Introduction

The continuous increase in anthropogenic CO_2_ emissions has become one of the most critical environmental challenges threatening global climate stability, ecosystem sustainability, and human health [1]. To achieve carbon neutrality and mitigate climate change, the development of efficient carbon capture, utilization, and storage (CCUS) technologies has been become an urgent global priority [2,3,4]. However, conventional amine-based solvents still suffer from several inherent drawbacks, including high volatility, severe corrosiveness, solvent degradation, and high regeneration energy consumption [5,6]. Therefore, the development of alternative carbon capture technology featuring high efficiency, low energy consumption, and long-term stability has attracted widespread attention [7].

**Figure 1:**
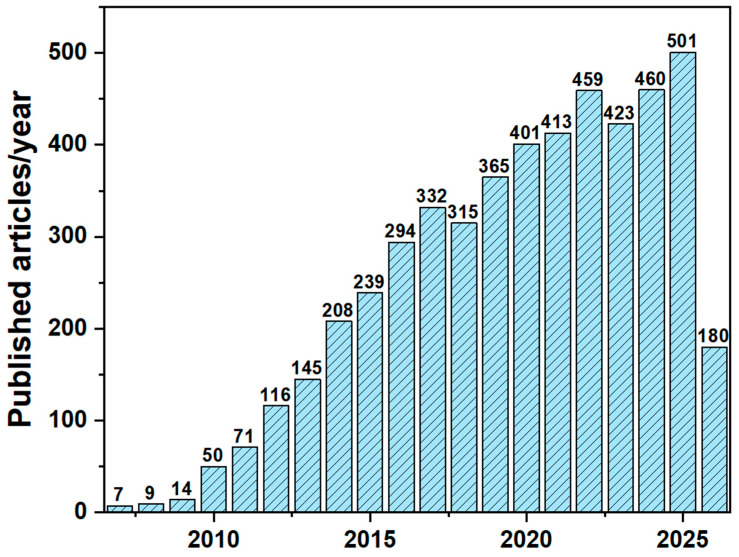
Articles published each year contain the keywords “CO_2_ capture” and “ionic liquids”.

Ionic liquids (ILs) are a class of organic salts entirely composed of cations and anions that typically remain liquid below 100 °C [8]. Owing to their negligible vapor pressure, excellent thermal and chemical stability, wide electrochemical windows, and highly tunable molecular structures, ILs have been widely regarded as promising media for CO_2_ capture [9,10,11]. By tailoring the types and functional groups of cations and anions, the polarity, free volume, hydrogen-bonding capability, and CO_2_ affinity of ILs can be precisely regulated at the molecular level, thereby endowing them with excellent CO_2_-selective adsorption performance, particularly under low CO_2_ partial pressure conditions [12,13]. As of 27 May 2026, a search of the Web of Science database using the keywords “CO_2_ capture” and “ionic liquids” indicates that this field is rapidly progressing toward greater performance (Figure 1). Nevertheless, despite their high tunability and superior CO_2_ adsorption selectivity, bulk ILs still suffer from several limitations, including intrinsically high viscosity, slow gas diffusion, severe mass-transfer resistance, and limited gas–liquid interfacial area [14,15,16]. These drawbacks not only hinder CO_2_ transport within ILs but also significantly increase the energy consumption of adsorption–desorption processes, thereby severely restricting their practical large-scale applications in carbon capture [13].

To overcome these limitations, researchers have increasingly incorporated ILs into porous materials to construct hybrid materials with both high CO_2_ affinity and rapid mass-transfer capability [17,18,19]. Porous materials generally refer to solid materials containing interconnected pores or cavities, including porous silica materials [20,21,22], porous carbon materials [23,24,25], metal–organic frameworks (MOFs) [26,27,28], and covalent organic frameworks (COFs) [29,30,31]. These materials possess large specific surface areas, tunable pore structures, abundant surface sites, and well-ordered porous channels, which not only provide rapid diffusion pathways for gas molecules but also enable the highly dispersed immobilization of ILs, thereby effectively reducing the mass-transfer resistance of bulk ILs and improving active-site utilization [32,33,34,35,36]. More importantly, the confined spaces created by nanopores can significantly alter the local microenvironment of ILs, causing confined ILs to exhibit physicochemical behaviors distinct from those of bulk ILs [37,38]. Compared with bulk ILs, ILs confined within porous structures often exhibit ionic rearrangement, free-volume variation, local electric-field modulation, and enhanced interfacial interactions [38,39]. These confinement-enhanced effects not only facilitate the diffusion and enrichment of CO_2_ within nanopores but also significantly improve CO_2_ capture performance by strengthening CO_2_–IL interactions, increasing active-site accessibility, and regulating adsorption thermodynamics [40,41,42,43].

Although numerous studies have been separately reviewed IL-based CO_2_ capture systems and porous adsorbent materials, systematic understanding and analysis of the confinement-enhanced mechanisms arising from the incorporation of ILs into porous materials remain insufficient [44,45,46,47]. Therefore, the primary objective of this review is to systematically elucidate the confinement-enhanced effects of ILs within porous materials, with particular emphasis on how nanoscale confinement regulates the free volume, pore structures, interfacial interactions, active-site accessibility, and adsorption thermodynamics of ILs. On this basis, the hybridization strategies of ILs with porous silica materials, porous carbon materials, MOFs, and COFs, as well as their applications in CO_2_ capture, are comprehensively summarized. Moreover, this review discusses the CO_2_ capture mechanisms, recent advances, and current limitations of bulk IL, thereby providing a theoretical basis for understanding the performance enhancement mechanisms in hybrid adsorbents. Furthermore, the life-cycle assessment (LCA), techno-economic analysis (TEA), and practical challenges associated with ionic liquid (IL)-based hybrid systems are critically analyzed, and future perspectives for the rational design and industrial application of confinement-enhanced IL hybrid adsorbents are proposed.

## 2. Applications and Challenges of ILs in CO_2_ Capture

**Figure 2:**
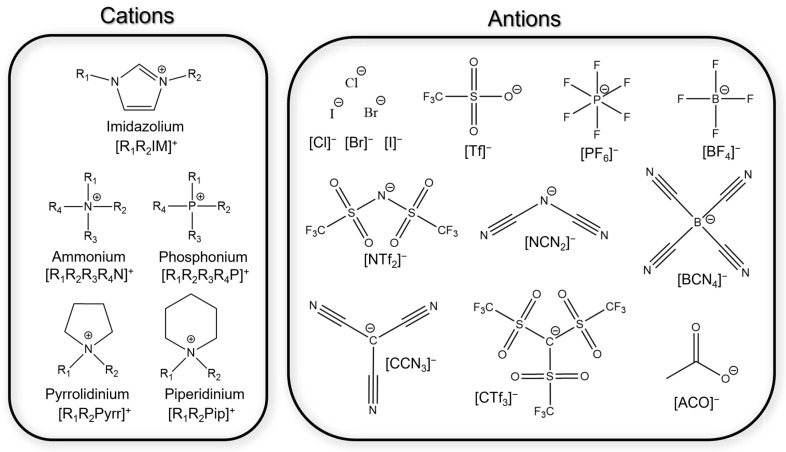
Commonly used anions and cations in ILs for CO_2_ capture.

The outstanding advantages of ILs in CO_2_ capture stem from their high degree of structural designability. By independently controlling the structures of cations and anions, their physicochemical properties can be precisely tailored [7,12]. Common cations and anions are shown in Figure 2.

### 2.1. CO_2_ Capture Mechanism by ILs

The interaction mechanism between ILs and CO_2_ primarily involves two types: physical adsorption and chemical adsorption. The specific mechanism depends on the molecular structure and functional group composition of the IL [44]. By controlling the alkyl side-chain length, introducing electron-donating or electron-withdrawing groups, and constructing functional sites such as -NH_2_, -OH, or -COO^−^, the polarity, free volume, and local interaction environment of ILs can be precisely tuned at the molecular level [13]. This, in turn, influences the interaction patterns and adsorption behavior between ILs and CO_2_.

#### 2.1.1. Physical Adsorption

**Figure 3:**
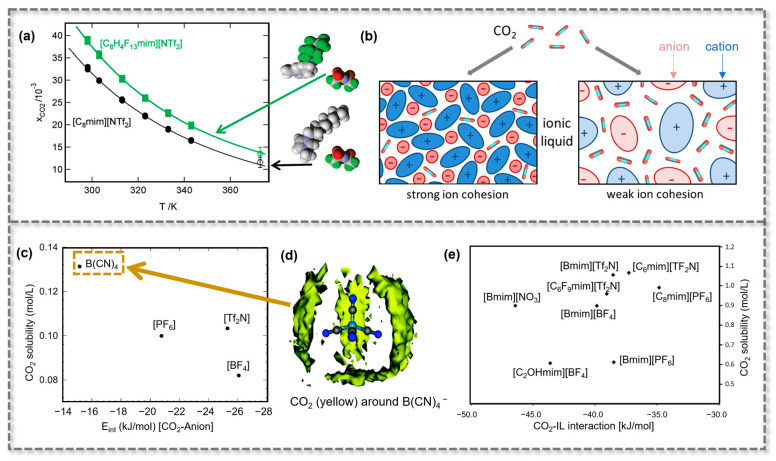
(**a**) Comparison of CO_2_ solubility as a function of temperature in [C_8_H_4_F_13_mim][NTf_2_] and [Omim][NTf_2_] [61]. (**b**) Schematic illustration of the influence of free volume on CO_2_ absorption in ILs [62]. (**c**) Correlation between CO_2_–anion interaction energy and CO_2_ solubility [63]. (**d**) Local distribution of CO_2_ in the [B(CN)_4_]^−^-based system [64]. (**e**) Relationship between cation–anion interaction energy and CO_2_ solubility [62].

Physical trapping primarily depends on non-covalent interactions between CO_2_ and ILs, including electrostatic interactions, van der Waals forces, and weak hydrogen bonding [48,49,50]. This process typically involves low adsorption enthalpy, leading to reduced energy consumption during desorption and solvent regeneration, while maintaining good reversibility and stability [51]. However, physical absorption capacity is relatively limited and sensitive to temperature, pressure, and the microstructure of the system [52,53,54]. Extensive studies indicate that the anion structure generally exerts a greater influence on CO_2_ solubility than the cation structure [55,56,57]. Anthony et al. systematically examined various cation systems and reported that ILs containing [NTf_2_]^−^ exhibit higher CO_2_ solubility than those incorporating [BF_4_]^−^ or [PF_6_]^−^ anions [58]. When the cation was fixed as [Bmim]^+^, CO_2_ solubility followed a clear anion-dependent trend: [NO_3_]^−^ < [DCA]^−^ < [BF_4_]^−^ ≈ [PF_6_]^−^ < [TfO]^−^ < [NTf_2_]^−^ < [methide]^−^ [59]. This trend is commonly attributed to the stronger polarizability of fluorinated anions and their enhanced weak Lewis acid–base and electrostatic interactions with CO_2_ [60]. Consistently, fluorinated cations can further increase CO_2_ solubility. For example, the perfluoroalkyl-substituted [C_8_H_4_F_13_mim][NTf_2_] exhibits higher absorption capacity than [Omim][NTf_2_] (Figure 3a) [61].

However, the strength of intermolecular interactions alone cannot fully account for the physical dissolution behavior of CO_2_ in ILs. The free-volume distribution, governed by interionic cohesive forces, also plays a critical role [62]. When interionic interactions are weak, the system more readily forms transient cavities capable of accommodating CO_2_, thereby enhancing solubility (Figure 3b). For example, despite its lower polarity, the [B(CN)_4_]^−^-based ILs exhibit higher CO_2_ solubility than the commonly studied [NTf_2_]^−^ system (Figure 3c) [63]. This behavior primarily arises from the weaker cohesive interactions between [B(CN)_4_]^−^ and the corresponding cations, leading to a looser structural organization and a significantly increased fractional free volume (FFV) (Figure 3d) [64]. Therefore, CO_2_ dissolution in IL is jointly governed by CO_2_–anion interactions and the free-volume effect (Figure 3e).

#### 2.1.2. Chemical Adsorption

**Figure 4:**
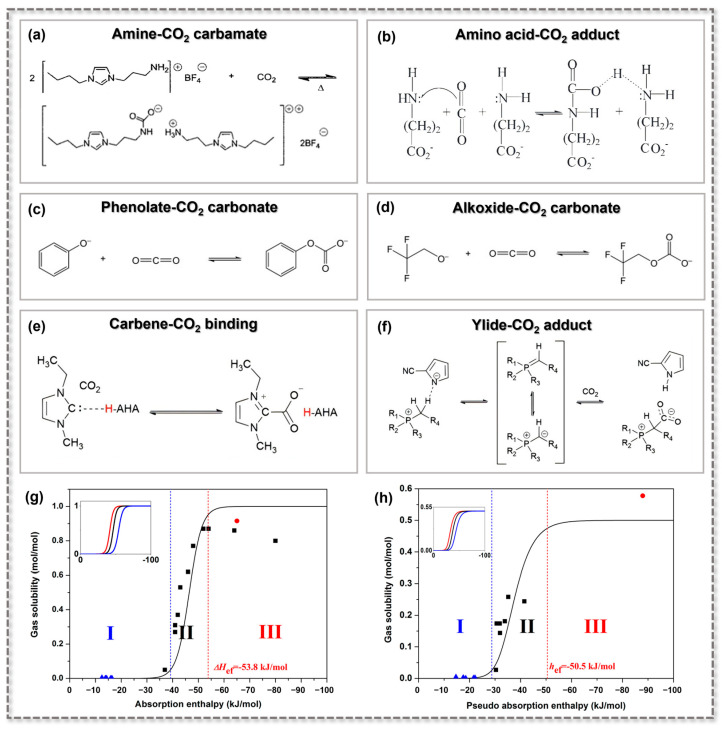
CO_2_ capture mechanisms in ILs: (**a**) Carbamate formation in primary-amine-functionalized ILs [69]. (**b**) Ammonium carbamate in amino acid anionic ILs [70]. (**c**) Carbonate formation in deprotonated phenolate ILs. (**d**) Carbonate formation in alkoxide ILs [72]. (**e**) Carbene generation via imidazolium deprotonation [13]. (**f**) Amphiphilic intermediates in phosphonium ILs [74]. (**g**) CO_2_ solubility vs. absorption enthalpy in 1:1 chemisorption ILs at 298 K and 0.15 bar (red line: T = 298.15 K and PCO_2_^G^= 1 bar; black line: T = 298.15 K and PCO_2_^G^ = 0.15 bar; blue line: T = 348.15 K and PCO_2_^G^ = 0.15 bar. Blue triangles: physical absorbents, including normal organic solvents and ionic liquids; black squares: chemically tunable ionic liquids with aprotic heterocyclic anions and amino acid anions; red circle: 25 wt % aqueous MDEA). (**h**) Absorption enthalpy vs. capacity in 1:2 chemisorption ILs at 298 K and 0.15 bar [81].

Compared with physical adsorption, chemical adsorption involves the formation of reversible chemical bonds between CO_2_ and functionalized ILs, which markedly enhances capture capacity and selectivity [65,66,67]. This approach is particularly suitable for post-combustion flue gas treatment and direct air capture (DAC) under low CO_2_ partial pressures. The capture performance of representative ILs is summarized in Table 1. The underlying mechanism is primarily based on nucleophilic attack facilitated by Lewis acid–base interactions, with active sites typically located on carbon, nitrogen, or oxygen atoms [13,44,68]. For instance, cations bearing primary amine groups or amino-acid-based anions can nucleophilically attack the carbon atom of CO_2_ via the nitrogen atom, forming carbamate or ammonium carbamate (Figure 4a,b) [69,70]. Due to hydrogen-bonding networks and conformational constraints, these systems generally exhibit a 1:2 stoichiometry, with a theoretical absorption capacity limited to 0.5 mol CO_2_ per mol of active site. Moreover, phenolate and alkoxide-type ILs can achieve chemical capture through O-site reaction with CO_2_ to form carbonates following deprotonation induced by superbases (Figure 4c,d) [71,72]. When the basicity of the anion is further increased, it can abstract an acidic proton from imidazolium or phosphonium cations to generate carbene or zwitterionic intermediates, allowing CO_2_ to bind directly to the carbon center in a 1:1 stoichiometry (Figure 4e,f) [65,66,73,74,75]. Although this pathway overcomes the stoichiometric limitation, it simultaneously introduces stronger conformational constraints.

Although chemisorption markedly strengthens the interaction between CO_2_ and ILs, its benefits are not unlimited. The thermodynamic feasibility of both physical and chemical absorption is governed by the Gibbs free energy change (ΔG) [76,77,78]. As interaction strength increases, the decrease in absorption enthalpy (ΔH) is typically accompanied by a greater entropy loss (−TΔS), thereby substantially increasing regeneration energy consumption [79,80]. Huang et al. identified a generalized S-shaped relationship between CO_2_ solubility and absorption enthalpy based on the van’t Hoff equation and thermodynamic equilibrium modeling (Figure 4g,h) [81]. Good agreement between experimental and model results was observed when |ΔH| < 40 kJ/mol. In this range, the system is dominated by physical dissolution (region I). Within the moderately exothermic regime, solubility becomes highly sensitive to ΔH (region II). Upon approaching the stoichiometric upper limit, the system enters a high-capacity plateau (region III), where further increases in |ΔH| scarcely enhance capacity but markedly raise regeneration energy demand. This trend applies to both 1:1 and 1:2 chemisorption systems, with plateau heights constrained by stoichiometric ratios. Notably, although stronger CO_2_–IL interactions can improve uptake capacity, they are generally accompanied by significant entropy penalty and higher regeneration energy requirements. Therefore, simply pursuing stronger binding is not optimal; instead, mitigating kinetic and energetic limitations while maintaining high selectivity is crucial for the continued development of IL CO_2_ capture systems.

**Table 1:** Summary of adsorption properties of selected ILs.

IL	P_CO2_ (bar)	T (K)	CO_2_ Absorption Capacity (mol CO_2_/mol IL)	Dominant Interaction Mechanism	Reaction Enthalpy (kJ/mol)	Ref.
[Hmim][NTf_2_]	8.59	298	0.31	Physisorption	-	[82]
[Hmim][BF_4_]	8.99	298	0.20	Physisorption	-	[82]
[Hmim][PF_6_]	9.27	298	0.20	Physisorption	-	[82]
[Emim][BF_4_]	8.75	298	0.12	Physisorption	-	[82]
[Emim][NTf_2_]	9.03	298	0.26	Physisorption	-	[82]
[Emim][TfO]	149	303	1.67	Physisorption	-	[83]
[Bmim][BF_4_]	10	303	0.07	Physisorption	-	[84]
[Bmim][PF_6_]	10	303	0.20	Physisorption	-	[84]
[Bmim][NTf_2_]	10	303	0.29	Physisorption	-	[84]
[Hmim][TfO]	11.5	304	0.38	Physisorption	-	[83]
[Omim][TfO]	12.5	304	0.40	Physisorption	-	[83]
[Bmim][NO_3_]	-	308	-	Physisorption	−16	[85]
[Bmim][SCN]	-	308	-	Physisorption	−12	[85]
[S_222_][NTf_2_]	19	313	0.44	Physisorption	−14.6	[86]
[Deme][NTf_2_]	19	313	0.47	Physisorption	−13.4	[86]
[Pmim][NTf_2_]	19	313	0.46	Physisorption	−10.5	[86]
[Amim][NTf_2_]	19	313	0.46	Physisorption	−14.3	[86]
[4Mbp][BF_4_]	19	313	0.26	Physisorption	−12.7	[86]
[ImNH_2_][BF_4_]	1	295	0.50	Primary amine	-	[69]
[P_66614_][4NH_2_-NC]	1	303	0.80	Primary amine	-	[87]
[18C6-K][Pro]	1	298	0.99	Secondary amine	−39	[88]
[MTBD][Im]	1	296	1.03	Tertiary amine	−85.2	[72]
[P_66614_][Triz]	1	303	0.95	Tertiary amine	−56.4	[89]
[P_66614_][Ind]	1	303	0.98	Tertiary amine	−52	[89]
[P_66614_][Im]	1	303	1	Tertiary amine	−49	[89]
[P_66614_][4-ABI]	1	293	1.60	Di-tertiary amine sites	-	[90]
[MTBDH][TFE]	1	296	1.13	Alkoxide	–16.8	[72]
[P_66614_][4-Me-PhO]	1	303	0.91	Phenoxide	-	[71]
[P_66614_][3-HMPz]	1	293	0.96	Phenoxide	−4.5	[91]
[P_66614_][PPhO]	1	293	0.93	Phenoxide	−51	[91]
[P_66614_][2-Op]	1	293	1.58	Synergistic chemisorption	-	[90]
[P_4442_][IDA]	1	313	1.69	Cooperative chemisorption	−89, −52.8	[92]

- Specific values are not mentioned in the cited articles; P_CO2_ (bar): Partial pressure of CO_2_ during the adsorption measurement.

### 2.2. Progress and Challenges of CO_2_ Capture Using ILs

The key breakthrough in the early development of IL research was the discovery of the high solubility of CO_2_ in typical ILs such as [Bmim][PF_6_] [93]. However, ILs based on physical absorption are primarily suitable for high-pressure conditions and exhibit limited efficiency under low-pressure environments, such as post-combustion carbon capture (PCC). To address this limitation, research has been progressively shifted toward functionalized ILs [94,95,96]. Introducing specific functional groups, such as amine [69,97], amino acid [70,98], carboxyl [99], pyridine [100], or imidazole moieties [13,101] into the IL structure enhances chemical reactivity with CO_2_, thereby significantly improving capture capacity and selectivity. For example, Bates et al. reported task-specific ILs featuring primary-amine-functionalized imidazolium cations [69]. Building on this concept, amino-acid-based ILs have attracted significant attention owing to their environmental compatibility, high CO_2_ uptake capacity, and good regenerability [102]. Subsequently, new IL systems were developed by combining propane-sulfonate-functionalized imidazolium cations with amino acid anions for CO_2_ capture [103]. However, many functionalized ILs exhibit a marked increase in viscosity after CO_2_ uptake due to hydrogen-bond network reorganization, which diminishes their high-capacity advantage [104,105].

To reduce viscosity while simultaneously enhancing CO_2_ uptake capacity, efforts have focused on the gradual introduction of electron-withdrawing and CO_2_-philic functional groups [106,107]. Research results have been shown that incorporating carbonyl-containing groups, such as -OAc or -CHO, into the anion is more effective than modifying the cation, as these groups can form stable complexes with CO_2_ via chemisorption [108]. For example, Chen et al. introduced electron-withdrawing groups, including IDA, HIDA, EDDA, and NTA, into amino acid anions to reduce the negative inductive effect of the amino group, thereby enhancing the affinity of carboxylates for CO_2_ [92]. Consequently, [P_4442_]_2_[IDA] achieved an absorption capacity as high as 1.69 mol CO_2_ per mol IL at 313 K and 1 bar. In addition, phenolate-, pyridinate-, and carboxylate-based ILs have attracted considerable attention owing to their relatively low viscosities [44,109].

To address the viscosity increase observed in protic ILs due to hydrogen-bonded complex formation after CO_2_ absorption, Wang et al. synthesized protic ILs via direct neutralization of superbases with weak proton donors [72]. Most systems achieved CO_2_ uptake values exceeding 1 mol CO_2_ per mol IL and remained liquid after absorption, without gelation, which is beneficial for mass transfer and process operation. In addition, Samuel Seo et al. proposed and systematically investigated a series of ILs based on aprotic heterocyclic anions by pairing [P_66614_]^+^ with azole-based heterocyclic anions (AHAs) derived from indazole, benzimidazole, and pyrrole [80]. Molecular dynamics simulations revealed that these AHA ILs exhibit nearly 1:1 stoichiometric CO_2_ uptake at 0–1 bar and 295 K. Owing to the absence of acidic protons, the number of hydrogen bonds changes minimally before and after CO_2_ uptake. Meanwhile, ionic translational and rotational dynamics remain essentially unchanged, resulting in negligible viscosity increase upon saturation [110].

Although this issue can be partially mitigated through molecular design, for example, by using superbase-derived protic ILs or non-protic heterocyclic anion systems [80,111,112], these strategies are typically associated with engineering challenges, including complex synthesis, high cost, and difficulty in scale-up [113].

## 3. Strategies for Hybridizing ILs with Porous Materials

To overcome mass-transfer limitations arising from the high viscosity of ILs, while fully leveraging their selective solubility and molecular recognition capabilities, ILs are incorporated into porous materials to form hybrid adsorbents [36]. The essence of this strategy lies in confining or anchoring ILs within nano- to sub-nanometer-scale pores via physical or chemical approaches, thereby enabling synergistic integration of the large surface area of porous supports with the functionalized interfaces of ILs. This, in turn, enhances both the kinetic and thermodynamic performance beyond that of conventional bulk IL [40,114].

### 3.1. Confined Effects and Interfacial Adsorption

Confinement of ILs within porous materials is not merely a filling process but a complex physicochemical transformation that fundamentally reshapes interfacial adsorption behavior. Unlike conventional adsorption mechanisms, which mainly rely on intrinsic surface sites or pore-filling effects, confinement-enhanced mechanisms arise from the synergistic interactions between confined ILs and nanoscale porous environments [40,41,114,115]. Under confinement, restricted spaces and interfacial interactions can reorganize IL microstructures, alter ion dynamics, regulate free volume, and reconstruct local physicochemical environments, thereby generating non-bulk properties unattainable in conventional adsorbents [38,39]. This mechanism differs fundamentally from traditional physical or chemical adsorption [116]. Conventional adsorption primarily depends on direct interactions between gas molecules and solid surfaces, with performance governed by factors such as surface area, pore size, and binding affinity [117]. In contrast, confinement-enhanced adsorption originates from the altered behavior of ILs within nanopores. The porous framework functions not only as a passive support but also as an active confinement environment that regulates IL configuration, transport behavior, and interfacial states [40,118]. As a result, the enhanced adsorption performance arises from the cooperative effects of pore confinement, IL restructuring, and interfacial interactions, rather than solely from the intrinsic adsorption capacity of the solid framework [39,41,114].

#### 3.1.1. Free-Volume Modulation of Confined ILs

**Figure 5:**
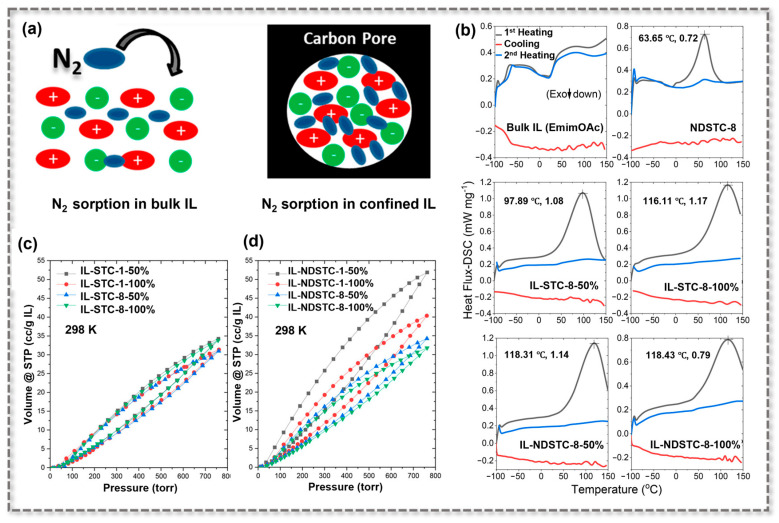
(**a**) Schematic illustration of free-volume enhancement in ILs under nanopore confinement. (**b**) Thermal fingerprints of confined ILs obtained from DSC analysis. (**c**,**d**) Volumetric N_2_ sorption isotherms and enhanced gas uptake behavior of confined IL systems [39].

When ILs are confined within porous materials such as zeolites, MOFs, porous carbons, and mesoporous silica, the pore structures of the host materials can significantly alter the microstructural organization of the ILs, with one of the most direct consequences being changes in free-volume fraction [43,116]. Nanoconfinement can weaken the strong ion–ion interactions that dominate in bulk ILs, thereby promoting an increase in free volume [38]. Harmanli et al. systematically investigated confined [Emim][OAc] within carbon pores and found that the mismatch between pore size and ion size forces the IL to adopt a “frustrated arrangement” under nanoconfinement [39]. This disrupts the extended and optimized ionic network present in bulk ILs, driving the ions toward more open packing configurations that reduce local packing density and increase accessible free volume (Figure 5a). Differential scanning calorimetry (DSC) results revealed that the characteristic phase-transition signal of bulk [Emim][OAc] completely disappeared after confinement within micropores (Figure 5b). The absence of phase-transition behavior indicates that the confined ions exist in a coordinatively unsaturated state due to strong interactions with the pore walls, implying enlarged interionic distances and increased free volume. Consequently, even in the absence of measurable open porosity, the confined IL exhibited substantially enhanced gas absorption capability, with absorption performance more than ten times higher than that of the bulk IL (Figure 5c,d) [38].

Surface effects within nanoscale pores can further modify the phase behavior and ion dynamics of ILs, leading to properties distinct from those of bulk systems. For example, in V-shaped confined pores, ILs exhibit reduced phase-transition temperatures and enhanced ion diffusivity, both of which contribute to further increases in effective free volume [119,120]. The enlarged free volume provides more transient cavities for CO_2_ accommodation, thereby enhancing physisorption capacity [121,122]. Meanwhile, the more open ionic configurations induced by confinement can improve the accessibility of active sites, such as the -NH_2_ groups in amino-acid-based ILs, thereby facilitating chemisorption kinetics [123].

#### 3.1.2. Pore Structure Reconstruction

**Figure 6:**
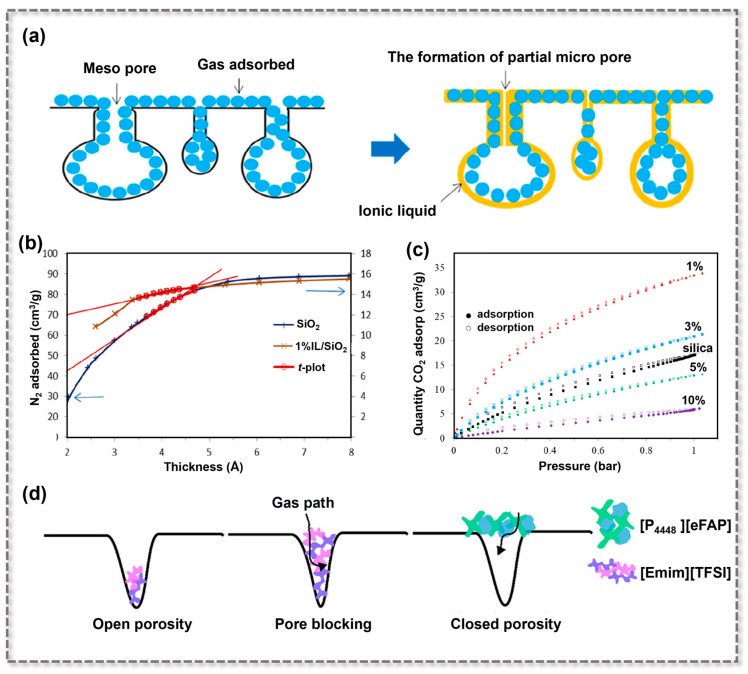
(**a**) Schematic illustration of partial micropore formation in the 1% [Bmim][CF_3_SO_3_]/SiO_2_ adsorbents. (**b**) t-Plot analysis correlating micropore volume with enhanced CO_2_ adsorption. (**c**) Comparison of CO_2_ physical adsorption isotherms at different IL loadings [118]. (**d**) Schematic representation of three typical distribution modes of ILs within microporous carbon [40].

ILs introduced into porous supports initially occupy the pore space, typically leading to pronounced decreases in specific surface area and pore volume [124,125,126]. For instance, in the [Bmim][CF_3_SO_3_]/SiO_2_ adsorbents prepared via a sol–gel method by Marliza et al., increasing the IL loading from 1% to 10% reduces the surface area from 266 m^2^/g to nearly 1 m^2^/g, accompanied by a substantial decline in pore volume [118]. More importantly, moderate loading does not merely cause pore blockage but can instead induce the formation of new microporous structures (Figure 6a). At a loading of 1%, however, the surface area decreases markedly, and the CO_2_ uptake increases from 33.7 to 66.7 mg/g. *t*-Plot analysis indicates that this enhancement arises from the partitioning and constriction of mesopores by a small amount of IL, generating micropores with sizes better matched to CO_2_ molecules (Figure 6b). This confinement-induced pore reconstruction effectively drives a transition from mesoporous to microporous structures, thereby strengthening physical adsorption (Figure 6c).

Furthermore, the size matching between IL molecules and pore dimensions dictates the pathway of pore structure evolution, as illustrated in Figure 6d [41,115]. Ayyildiz et al. demonstrated that, when the IL size is well matched with the pore diameter, ILs can penetrate pores and maintain an open-pore structure after partial filling [40]. In contrast, when the pore size is slightly smaller, ILs preferentially adsorb at the pore entrances and, at higher loadings, lead to the formation of closed pore structures. For bulkier ILs, a surface coverage layer is more likely to form at the pore openings, inducing pore blockage. This configuration effectively creates an “IL gate” at the pore exterior, allowing CO_2_ to diffuse through while imposing additional mass-transfer resistance on other gases, thereby enhancing selectivity to a certain extent.

#### 3.1.3. Interfacial Stabilization and Activation

Confining functionalized ILs within porous materials can markedly enhance the stability and utilization efficiency of active sites [127,128,129]. On the one hand, nanoscale confinement suppresses IL leakage and aggregation through capillary forces and interfacial interactions [130,131]. Wang et al. employed the Young–Laplace equation to theoretically predict and experimentally validate the mechanical stability of ILs within porous networks [42]:

(1) $$Ρ=\frac{2\gamma \cos\theta }{r_{p}}$$

**Figure 7:**
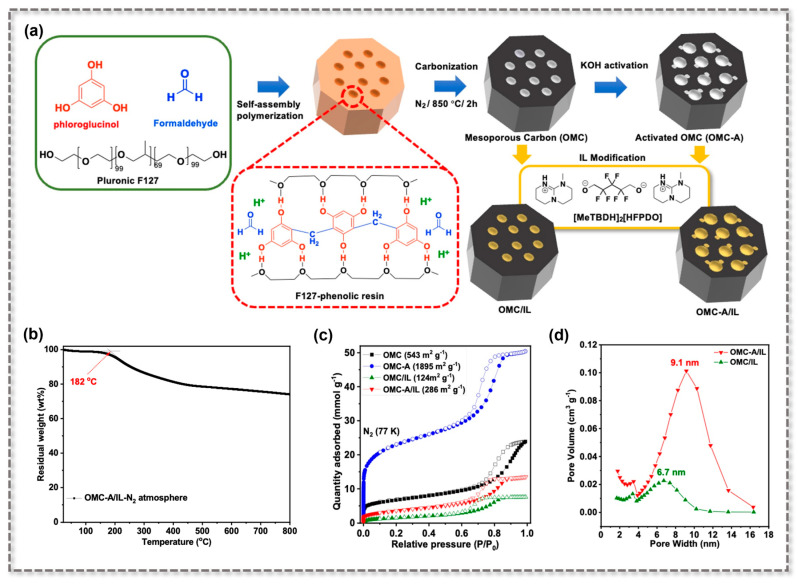
(**a**) Schematic of the synthesis of OMC and KOH-activated carbon supports. (**b**) Thermogravimetric comparison of the [MeTBDH]_2_[HFPDO] and the IL hybrid material confined within OMC-A pores. (**c**) Pore structure characterization of pristine OMC, OMC-A, and their IL-modified counterparts (solid symbols: adsorption branch; hollow symbols: desorption branch). (**d**) Mesopore size distribution of the OMC/IL and OMC-A/IL hybrid materials [41].

Here, the stability of ILs within pores is described by the breakthrough pressure (Equation (1)), which is positively correlated with surface tension (γ) and wettability (cos θ) and inversely proportional to the pore radius (r_p_). Accordingly, reducing pore size, increasing IL surface tension, and optimizing wettability can significantly enhance mechanical stability. Wang et al. further confined [Emim][Gly] within a single-walled carbon nanotube network, with both theoretical and experimental results indicating a breakthrough pressure exceeding 25 bar, maintaining structural and functional stability even under high-pressure gas conditions. On this basis, the confined effect is further reflected in enhanced thermal stability [129,132,133]. For example, Mokhtari-Nori et al. confined [MeTBDH]_2_[HFPDO] within ordered mesoporous carbon, which significantly improves the thermal stability of the IL, due to the synergistic effects of π–π interactions with the carbon materials and spatial confinement (Figure 7a) [41]. Thermogravimetric analysis shows that the onset decomposition temperature of the bulk IL is approximately 455 K, whereas it increases markedly after incorporation into OMC-A, indicating that confinement effectively suppresses thermally induced migration and decomposition (Figure 7b). Meanwhile, the pore structure remains well preserved, with a mesopore size of about 9.1 nm and a total pore volume of 0.91 cm^3^/g, ensuring continuous gas transport pathways (Figure 7c,d). Therefore, the rational construction of hierarchical pore structures can maintain efficient mass transfer while enhancing stability.

**Figure 8:**
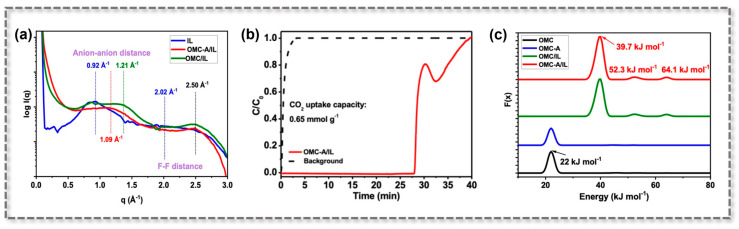
(**a**) Microstructural comparison between the bulk IL and the IL confined within carbon pores. (**b**) Dynamic CO_2_ adsorption of OMC-A/IL at 400 ppm CO_2._ (**c**) Energy distribution profiles of pristine carbon supports and IL-confined hybrid materials [41].

Beyond stabilization, confinement further strengthens CO_2_–IL interactions through interfacial regulation [41,114]. Compared with bulk ILs, those confined in nanopores undergo significant reorganization in local electric fields, molecular orientation, and electron distribution [39,50]. Confinement enhances electrostatic interactions between the CO_2_ quadrupole and ionic species, while modulating the interfacial electric field, thereby stabilizing adsorption intermediates and strengthening interactions, as demonstrated by Mokhtari-Nori et al. [41,134,135]. Wide-angle X-ray scattering shows that the characteristic peaks shift to higher scattering vectors after confinement, indicating a more compact packing structure within carbon nanopores (Figure 8a). This arises from strong interactions between the π-conjugated carbon surface and aromatic IL cations, leading to reorganization of molecular arrangement and electronic structure. Small-angle neutron scattering further reveals confinement-induced ordering, reflected by a narrower mesopore size distribution after IL incorporation and a reduced power-law decay upon CO_2_ adsorption. These results indicate a more ordered IL phase and a smoother interface under CO_2_, promoting greater exposure of active sites. Compared with conventional OMC, OMC-A, with larger surface area and larger mesopores, exhibits higher CO_2_ uptake at the same IL loading of 30 wt% (Figure 8b). This improvement is attributed to a more developed mesoporous network that enables uniform IL dispersion, thereby maximizing active-site utilization under dilute CO_2_ conditions [136].

Moreover, confined effects typically reduce the apparent reaction enthalpy of CO_2_ adsorption, as the interactions with the porous material surface moderately decrease the electronic density of active sites (Figure 8c), an effect akin to “interfacial passivation”. While this slightly weakens the binding strength at individual sites, it enhances thermodynamic reversibility, facilitates regeneration under mild conditions, lowers energy consumption, and improves cycling stability, thus achieving an optimized balance between adsorption capacity and energy efficiency [137,138,139]. Meanwhile, the introduction of hydrophobic ILs, such as fluorinated or long-alkyl-chain ILs, can create hydrophobic interfaces within pores or on surfaces, effectively suppressing water intrusion into active sites and mitigating competitive adsorption and structural hydrolysis [127,140]. For example, fluorinated IL-modified UiO-66 retains over 90% of its initial CO_2_ adsorption capacity at a relative humidity of 80% [141]. These observations suggest that synergistic control of pore structure and IL confinement is key to achieving efficient CO_2_ capture and separation.

### 3.2. Methods of Nanoscale Confinement

ILs typically interact with porous materials in two ways: first, via chemical coupling, where ILs are grafted onto pore walls through covalent bonds [142,143,144]; second, via physical impregnation, where ILs are encapsulated within the porous structure [145,146,147]. The adsorption capacity, selectivity, mass transfer kinetics, and regeneration energy of hybrid adsorbents can be finely tuned by synergistically controlling IL composition, pore structure, and loading mode, thanks to the designability of ILs and the tunable features of porous carriers [148,149]. Based on the combination mode of ILs with porous materials, current nanoscale confined strategies can generally be classified into two categories: in situ construction and post-synthetic modification.

#### 3.2.1. In Situ Construction Strategy

**Figure 9:**
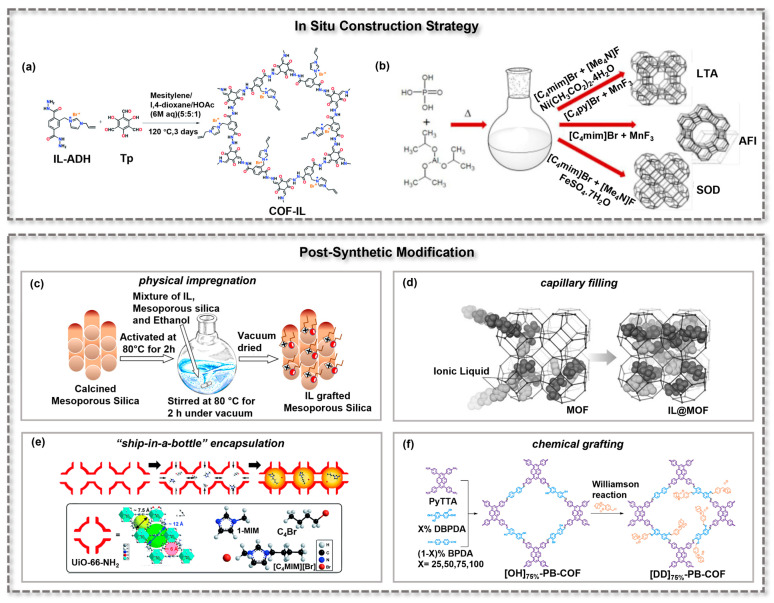
(**a**) Schematic illustration of the synthetic pathway and structural integration of ILs within COFs [142]. (**b**) IL-directed crystallization pathways of MAPOs toward AFI-, LTA-, and SOD-type framework topologies [143]. (**c**) Preparation of IL-loaded mesoporous silica via impregnation [145]. (**d**) Capillary-driven infiltration of [Emim][TFSA] into the micropores of ZIF-8 [146]. (**e**) MOF cage-enabled IL encapsulation via a stepwise “ship-in-a-bottle” strategy in UiO-66-NH_2_ [147]. (**f**) Schematic illustration of the synthesis of [DD]_X–_PB-COF (X = 25, 50, 75, 100) under the regulation of varying compositional ratios [144].

The in situ construction strategy involves introducing ILs or their precursors during the formation of porous materials. This allows them to directly participate in self-assembly, crystallization, or crosslinking processes, thereby enabling tighter structural synergy and interfacial coupling (Figure 9a,b) [150]. A defining feature of this approach is that ILs function not only as active components but also as reaction media, structure-directing agents, or catalytic species, regulating pore architecture and the local chemical environment at the molecular level [143,151]. Under solvothermal or ionothermal conditions, ILs can cooperatively modulate framework formation through electrostatic interactions, steric effects, and weak coordination interactions with inorganic or organic building units [152,153]. This method results in the stable confinement of IL species within pores or cage-like structures. Such hybrid adsorbents typically exhibit enhanced loading stability and a more uniform distribution of active sites, which is advantageous for efficient CO_2_ capture under low partial pressure. Furthermore, constructing polymeric IL networks via in situ polymerization or crosslinking can further suppress IL migration while improving the thermal stability and cyclic durability of the hybrid materials [154].

#### 3.2.2. Post-Synthetic Modification

Post-synthetic modification involves introducing ILs into pre-formed porous materials through physical impregnation [145,155,156], capillary filling [146,157,158,159], “ship-in-a-bottle” encapsulation [147,160,161,162], or chemical grafting [144,163], constructing hybrid structures via pore-confined effects and interfacial interactions (Figure 9c–f). This strategy is mild, operationally flexible, and compatible with diverse porous materials, making it one of the most widely applied hybridization approaches [164]. In physical modification, ILs are typically distributed within pores as ultrathin liquid films or nanodroplets, with loading primarily governed by van der Waals forces, hydrogen bonding, and capillary effects [146,155,157,159]. This method is straightforward but may encounter IL migration or leakage under high-loading conditions. In contrast, “ship-in-a-bottle” encapsulation and chemical grafting achieve spatial confinement or strong interfacial anchoring of ILs through size matching or covalent bonding, effectively preventing leakage and deactivation during operation and significantly enhancing cycling stability [144,147,160,163]. Table 2 summarizes the advantages and limitations of various hybridization strategies for comparative reference.

As shown in Table 2, different hybridization strategies involve inherent trade-offs among IL loading, pore accessibility, and long-term stability. Among these factors, IL migration, leakage, or structural deactivation during repeated adsorption–desorption cycles remains a challenge affecting the long-term performance and practical applicability of hybrid materials [144,163,168]. Overall, in situ construction strategies are generally more effective in improving IL immobilization stability, as they enable stronger interfacial coupling and structural synergy during material formation [150]. In contrast, post-synthetic modification strategies primarily rely on pore confinement effects, capillary forces, or chemical bonding to achieve stable IL anchoring [155,159]. Among these approaches, “ship-in-a-bottle” encapsulation and chemical grafting are particularly effective in suppressing IL leakage and enhancing cycling stability [147,160].

Moreover, constructing polymeric IL networks or strengthening interfacial interactions can further reduce IL migration, thereby improving the stability and reusability of hybrid materials under harsh operating conditions [169,170,171]. On the one hand, the intrinsic stability of ILs can be enhanced through structural optimization. For example, polyprotic ILs containing multiple –OH, or –SO_3_H groups within the cation not only strengthen interactions with target gases but also reduce IL migration and loss during regeneration through enhanced intermolecular interactions [168]. On the other hand, introducing additional functional components can further improve the robustness of hybrid materials. For instance, bimetallic IL-hybridized zeolite adsorbents and amino-functionalized porous ILs provide additional interaction sites through synergistic multicomponent effects, thereby maintaining good cycling stability under harsh conditions [172].

## 4. Applications of IL Hybrid Materials in Carbon Capture

ILs can be coupled with a variety of porous materials to construct hybrid adsorbents with diverse performance characteristics. Common supports include porous silica-based materials [22,173,174,175], porous carbon materials [176,177,178], MOFs [46,179,180,181,182], and COFs [183,184,185]. These materials exhibit clear differences in pore architecture, specific surface area, surface chemistry, and mechanical and chemical stability (Table 3), which in turn determine their interaction modes with ILs, achievable loading capacities, and the resulting mass-transfer and adsorption behaviors [186,187,188,189,190]. The following sections systematically discuss the design strategies for these IL/porous hybrid materials, as well as their applications in carbon capture.

### 4.1. IL/Porous Silicon Hybrid Materials

**Figure 10:**
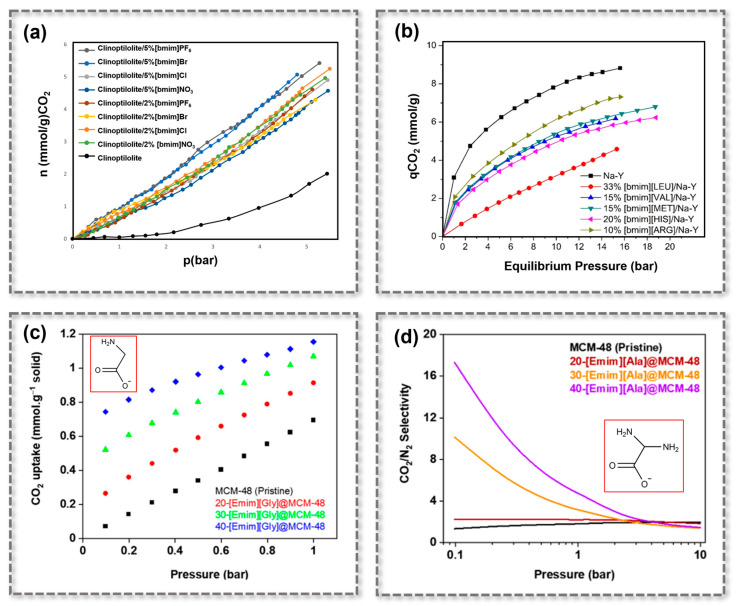
(**a**) CO_2_ uptake of [Bmim]^+^ ILs at various pressures. (**b**) CO_2_ adsorption isotherms of Na-Y and amino-acid-IL-loaded Na-Y [186]. (**c**) CO_2_ adsorption isotherms of [Emim][Gly]@MCM-48 hybrid materials with different loadings at 303 K. (**d**) CO_2_/N_2_ selectivity of [Emim][Ala]@MCM-48 hybrid materials with different loadings at 313 K [191].

Porous silicon-based materials include microporous zeolites and ordered mesoporous silica, which differ in pore size and surface chemistry, leading to distinct IL-confined and CO_2_ capture behaviors [191,192,193]. Microporous zeolites, such as 13X and Na-Y, feature uniform sub-nanometer channels and exchangeable metal cations, enabling high CO_2_ selectivity via Lewis acid–base interactions and molecular sieving [194,195]. Mortazavi et al. incorporated 2–5 wt% [Bmim]^+^ ILs into natural clinoptilolite via wet impregnation [186]. The 5 wt% [Bmim][PF_6_] sample exhibited a 4.35-fold higher CO_2_ uptake than pristine zeolite at 4 bar and 298 K (Figure 10a), due to enhanced PF_6_^−^-CO_2_ interactions and highly dispersed IL within the pores, which improved IL–CO_2_ interfacial contact [134,196,197]. However, the sub-nanometer pores (<1.2 nm) of microporous zeolites lead to pore blockage and diffusion limitations at higher IL loadings [198]. Sistla and Khanna reported that IL loadings above 5 wt% severely disrupt Na-Y’s pore structure [191]. For example, 3% [Bmim][LEU]/Na-Y saw its specific surface area decrease from 650 m^2^/g to near zero, resulting in overall CO_2_ uptake lower than pristine zeolite (Figure 10b). Nevertheless, IL-specific CO_2_ capture increased markedly; in 33% [Bmim][LEU]/Na-Y, the IL-specific uptake was approximately three times that of neat IL. These findings indicate that, in strongly confined microporous systems, zeolites primarily enhance IL intrinsic adsorption by shortening diffusion paths and modulating the local microenvironment, rather than by providing additional physical adsorption sites.

In contrast, ordered mesoporous silica, such as MCM-41, SBA-15, and MCM-48, features large pores (2–50 nm), low diffusion resistance, and abundant silanol groups, providing an ideal platform for highly dispersed ILs [199,200]. Philip and Henni loaded [Emim][Gly] and [Emim][Ala] into the 3D cubic channels of MCM-48 via wet impregnation, achieving uniform IL distribution and rapid CO_2_ diffusion [192]. Amino acid anions react with CO_2_ via -NH_2_ to form carbamates, governing chemisorption. At 303 K and 0.1 bar, 40 wt% [Emim][Gly]@MCM-48 exhibited a CO_2_ uptake of 0.74 mmol/g, ten times that of pristine MCM-48 (Figure 10c), owing to small side-chain H atoms that enable high amine density. The bulkier methyl group of [Emim][Ala] reduces surface area and suppresses N_2_ adsorption at the same loading. Nevertheless, CO_2_ chemisorption remains effective, yielding a CO_2_/N_2_ selectivity of 17 for 40 wt% [Emim][Ala]@MCM-48, far surpassing that of pristine MCM-48 (Figure 10d).

**Figure 11:**
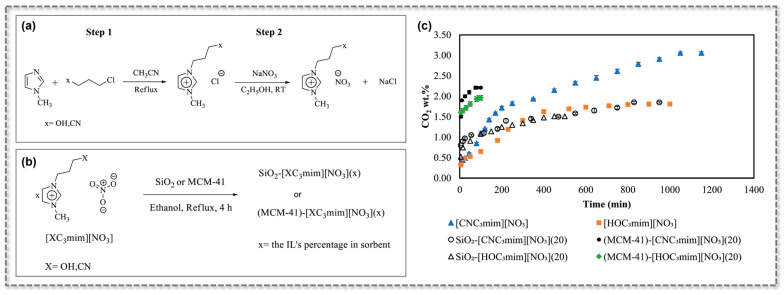
(**a**) Synthetic routes of ILs [OHC_3_mim][NO_3_] and [CNC_3_mim][NO_3_]. (**b**) Schematic of SiO_2_-IL(x) and MCM-41-IL(x) hybrid adsorbent preparation. (**c**) CO_2_ adsorption performance of different adsorbents at 298 K [201].

Similarly, Mirzaei compared the CO_2_ adsorption of hydroxyl-([OHC_3_mim][NO_3_]) and cyano-functionalized ([CNC_3_mim][NO_3_]) loaded on amorphous SiO_2_ and MCM-41 (Figure 11a,b) [201]. The cyano-group significantly enhanced CO_2_ affinity, with MCM-41-[CNC_3_mim][NO_3_] achieving 2.21 wt% uptake at 298 K and 1 bar, exceeding the 1.96 wt% observed for the hydroxyl analog (Figure 11c), due to the -CN group acting as a Lewis base. Consistent with the findings of Philip and Henni, increasing IL loading reduced surface area and N_2_ physisorption but improved CO_2_ selectivity. For instance, increasing [OHC_3_mim][NO_3_] loading from 10% to 40% raised the CO_2_/N_2_ selectivity of SiO_2_-based adsorbents from 15 to 25. These results indicate that functional group tuning combined with optimized IL loading enables mesoporous silica-based hybrid adsorbents to achieve both high CO_2_ uptake and selectivity.

**Figure 12:**
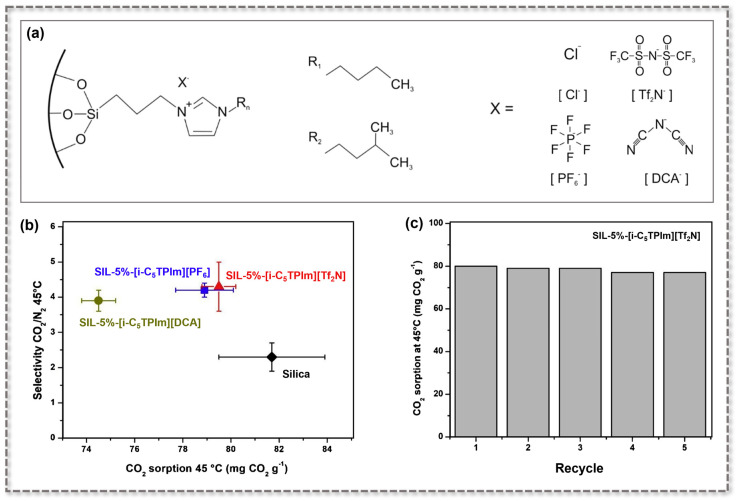
(**a**) Schematic of chemically grafted IL/mesoporous silica hybrid materials. (**b**) Effect of anion type on CO_2_ uptake and CO_2_/N_2_ selectivity of SIL-5–[i-C_5_TPIm]^+^ hybrids. (**c**) CO_2_ adsorption cycling stability of SIL-5–[i-C_5_TPIm][NTf_2_] hybrids [187].

To achieve efficient utilization and long-term stability of IL functional sites, chemical grafting has become an effective strategy for constructing mesoporous silica/IL hybrids [200]. In this approach, silane-functionalized IL cations are covalently anchored to surface silanol groups, ensuring uniform, high-density immobilization and suppressing IL leaching during operation. Duczinski et al. grafted -Si(OCH_2_CH_3_)_3_-functionalized imidazolium ILs ([C_4_TPIm][Cl], [i-C_5_TPIm][Cl]) onto mesoporous silica (Figure 12a) and tuned CO_2_ affinity through anion exchange [187]. At low loadings of approximately 5 wt%, pore accessibility is maintained while maximizing functional-site availability, resulting in CO_2_ uptake comparable to pristine silica. SIL-5%-[i-C_5_TPIm][NTf_2_] achieved 79.50 mg/g, compared with 81.70 mg/g for bare silica. Fluorinated anions such as [NTf_2_]^−^ and [PF_6_]^−^ double CO_2_/N_2_ selectivity at low loadings owing to strong CO_2_ affinity, large free volume, and the high electronegativity of fluorine, without compromising capacity (Figure 12b). Moreover, branched cation chains, for example, isopentyl [i-C_5_mim]^+^ versus linear butyl [mim]^+^, further increase free volume and enhance selectivity. Cycling tests confirm the excellent regeneration stability of these grafted hybrids (Figure 12c).

### 4.2. IL/Porous Carbon Hybrid Materials

**Figure 13:**
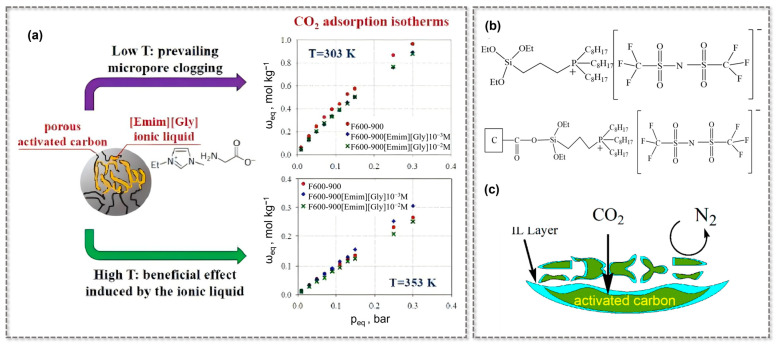
(**a**) Effect of temperature on the CO_2_ adsorption mechanism of [Emim][Gly]-loaded AC [173]. (**b**) Molecular structures of phosphonium-based ILs and a schematic illustration of their grafting onto AC. (**c**) Schematic diagram illustrating CO_2_/N_2_ selective separation achieved by an IL thin film on the surface of AC [210].

Porous carbon materials, including activated carbon (AC) [202,203], graphene [204,205], carbon nanotubes (CNTs) [206,207], and carbon molecular sieves, are widely utilized for CO_2_ capture owing to their well-developed pore structures, large surface areas, and excellent stability [208,209]. Among them, AC is particularly attractive due to its low cost and broad availability. Erto et al. loaded the physically adsorbing [Hmim][BF_4_] and the chemically reactive [Emim][Gly] onto two ACs with markedly different pore architectures [188]. The results indicate that ILs preferentially occupy micropores, leading to significant pore blockage, especially in the micropore-rich F600-900 AC sample, and consequently lower CO_2_ uptake for all modified samples compared with pristine carbons at 303 K (Figure 13a). When the temperature was increased to 353 K, the -NH_2_ group of the glycinate anion in [Emim][Gly] chemically interacted with CO_2_. At low IL loadings, the modified samples outperformed the unmodified carbons on both supports, demonstrating that introducing chemisorption sites while preserving pore accessibility is critical for performance enhancement. [Hmim][BF_4_], lacking reactive functional groups, was unable to compensate for the capacity loss caused by micropore blockage, even at elevated temperatures.

To address the limitations of micropore blockage on IL performance, He et al. compared impregnation and grafting methods for loading [P_8883_][NTf_2_] onto AC, as shown in Figure 13b [210]. The results showed that the impregnation method tends to form a relatively thick IL film, which enhances selectivity but severely obstructs the pore channels (Figure 13c). In contrast, grafting preserves the pore structure even at low IL loadings, enabling higher adsorption capacity and faster mass transfer.

**Figure 14:**
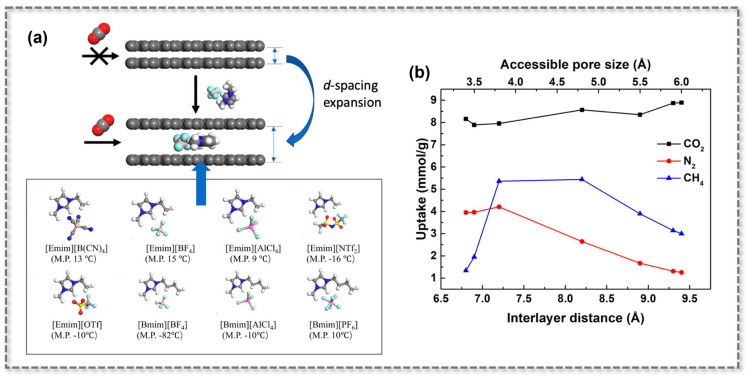
(**a**) Schematic design of graphene/IL hybrid materials for gas adsorption. (**b**) Adsorption capacities of CO_2_, N_2_, and CH_4_ at 298 K and 1 bar under different interlayer spacings and the corresponding accessible pore sizes [141].

From the above studies, it is clear that severe micropore blockage induced by IL incorporation in AC limits pore accessibility and mass transfer. Graphene, with an open, two-dimensional architecture, alleviates pore blocking and enables well-defined interlayer channels [211]. At the same time, its structural robustness and confinement capabilities make it an ideal matrix for stabilizing ILs in membrane-based systems [212,213,214]. In regulating the interfacial microenvironment through anion engineering, Wang et al. inserted ILs as interlayer spacers between graphene sheets to construct tunable slit-like pores [141]. Density functional theory (DFT) calculations and grand canonical Monte Carlo (GCMC) simulations revealed that the accessible pore size is primarily determined by the anion size, following the order [BF_4_]^−^ < [PF_6_]^−^ < [TfO]^−^ < [AlCl_4_]^−^ ≈ [NTf_2_]^−^ < [B(CN)_4_]^−^ (Figure 14a). Because the imidazolium cations adopt a flat orientation between graphene layers, their contribution to the interlayer spacing is limited. Therefore, the anions dominate the regulation of both the interlayer distance (6.8–9.4 Å) and the accessible pore size (3.4–6.0 Å). Adsorption simulations indicate that CO_2_ uptake slightly increases with pore size, whereas N_2_ adsorption decreases significantly. In contrast, CH_4_, whose molecular diameter of approximately 4.0 Å matches the pore size, exhibits a maximum adsorption capacity at this dimension due to optimal van der Waals interactions with the upper and lower graphene layers (Figure 14b). These results demonstrate that anion engineering enables nanoscale pore-size tuning, thereby optimizing CO_2_/N_2_ and CO_2_/CH_4_ separation performance and offering a promising strategy for the rational design of highly selective carbon capture materials.

**Figure 15:**
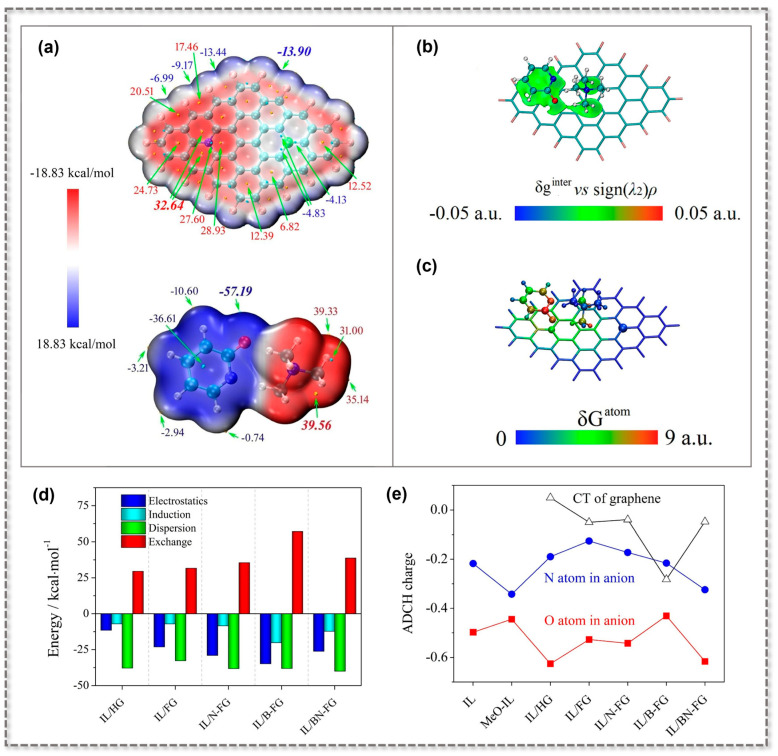
(**a**) Electrostatic potential distributions of BN-FG and [TMA][HPy]. (**b**) IGMH visualization of weak interactions between BN-FG and [TMA][HPy]. (**c**) Atom-colored δG^atom^ analysis of weak interactions in the BN-FG/[TMA][HPy] system. (**d**) Influence of different graphene supports on the binding energy of the [TMA][HPy] ion pair, along with energy decomposition analysis. (**e**) Synergistic modulation of anionic active-site charges induced by BN-FG [215].

Building upon the above work, Zhang et al. elucidated how graphene supports regulate the intrinsic properties of ILs from an electronic structure perspective [215]. Distinct from the geometrical pore modulation via interlayer confinement reported by Wang et al., they showed that fluorinated and heteroatom-doped graphene can directly modulate cation–anion interactions through surface electrostatic potential redistribution and charge transfer, while simultaneously reshaping the charge distribution at anionic active sites to enhance intrinsic CO_2_ affinity (Figure 15a). DFT calculations were conducted to systematically examine the interfacial interactions and electronic responses of the hydroxypyridine-based [TMA][HPy] on hydrogen-terminated graphene (HG), fluorinated graphene (FG), and boron–nitrogen co-doped fluorinated graphene (BN-FG). Among these, BN-FG exhibits the strongest modulation effect, markedly weakening hydrogen-bonding and coulombic interactions between ion pairs, with the binding energy decreasing from −100.42 to −81.89 kcal/mol, thereby promoting a shift from tightly associated to more dissociated ion pairs (Figure 15b,d). This is accompanied by a simultaneous increase in negative charge density at both N and O sites of the anion, enhancing the electron-donating capability of CO_2_ binding sites. This behavior contrasts with conventional anion substitution strategies, which typically involve a trade-off in charge distribution between N and O sites. Conversely, the doped graphene system enables concurrent enhancement at both sites, improving adsorption activity while maintaining a balanced charge distribution (Figure 15c,e).

**Figure 16:**
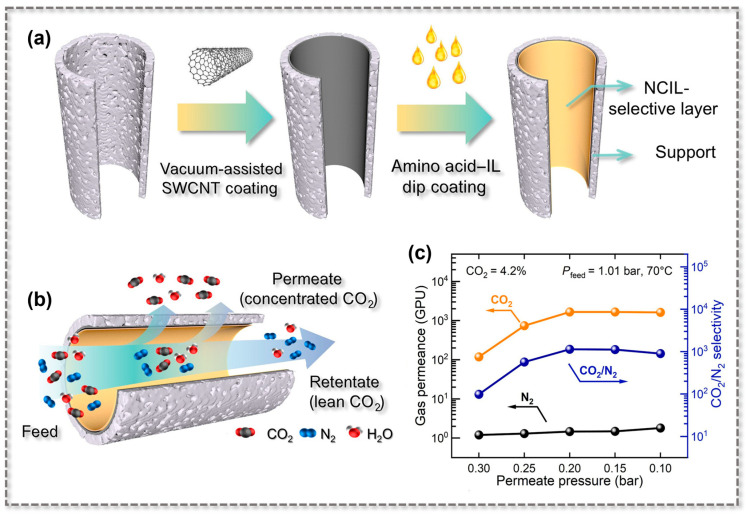
(**a**) Schematic illustration of the fabrication process of the nanoconfined IL hollow fiber membrane. (**b**) Schematic of the CO_2_ selective transport mechanism in the nanoconfined IL hollow fiber membrane. (**c**) Effect of permeate-side pressure on the separation performance of the nanoconfined IL membrane [42].

In addition, CNTs, composed of rolled graphene sheets, exhibit excellent mechanical properties and can efficiently capture CO_2_ over a temperature range of 273–473 K, with adsorption capacities nearly twice those of AC, making them attractive for gas adsorption under specific conditions [216,217,218]. Zeng et al. systematically investigated the CO_2_ adsorption and separation performance of IL-modified CNTs, with particular focus on stability under humid conditions [219]. The study revealed that water molecules form hydrogen bonds with the IL and interact with the CNT surface, occupying adsorption sites. Water uptake increases with pressure, leading to a significant decrease in CO_2_ adsorption at high pressures. Against this backdrop, Wang et al. proposed and fabricated a nanoconfined IL membrane composed of a single-walled CNT nanoscale confined network combined with highly selective amino acid ILs (Figure 16) [42]. By constructing an ultrathin transport layer with controllable thickness, open structure, and strong confined effects, the membrane achieves significantly enhanced CO_2_ permeance of up to 1654 GPU and CO_2_/N_2_ selectivity of up to 1132, while effectively addressing the liquid loss and poor stability issues that affect conventional supported IL membranes under high pressure and long-term operation. Although CNTs and graphene demonstrate outstanding CO_2_ adsorption performance, their strong intermolecular cohesion results in high separation and purification costs, limiting some of the industrial applications [220]. Therefore, further optimization of material synthesis and operational protocols is required to enable practical industrial deployment.

### 4.3. IL/MOF Hybrid Materials

MOFs, constructed from inorganic metal nodes and multidentate organic ligands, possess high crystallinity, ultrahigh surface area, tunable pore structures, open metal sites, and abundant functional sites, making them highly promising materials for CO_2_ adsorption and separation [182,221]. Representative structures such as MIL-53 [222,223], ZIF-8 [129], HKUST-1 [224], MOF-74 [225,226], and UiO-66 [227] have demonstrated considerable CO_2_ capture performance. However, pristine MOFs still face three major limitations in CO_2_ capture, particularly under low partial pressure conditions. First, many MOFs exhibit high water sensitivity; H_2_O can competitively occupy open metal sites or induce framework hydrolysis, thereby reducing adsorption capacity and cyclic stability [228,229]. Second, in some MOFs, rotatable ligand groups (e.g., -CH_3_) allow pore expansion during adsorption, weakening the molecular sieving effect for CO_2_/N_2_ separation [230]. Third, most MOFs rely primarily on physisorption, and their interaction differences with N_2_ and CH_4_ are relatively small, leading to a sharp decline in adsorption capacity under DAC conditions (Table 4, entries 1–3) [231]. Due to the designability of IL structure, introducing ILs with carbon capture sites into MOFs via impregnation, confinement, or grafting has been regarded as an effective strategy to enhance carbon capture performance [232,233]. This incorporation can improve CO_2_ adsorption by regulating pore structure and interfacial interactions (Table 4, entries 4–11). In addition, longer alkyl chains impart greater hydrophobicity, thus improving the material’s water vapor tolerance [234,235].

**Figure 17:**
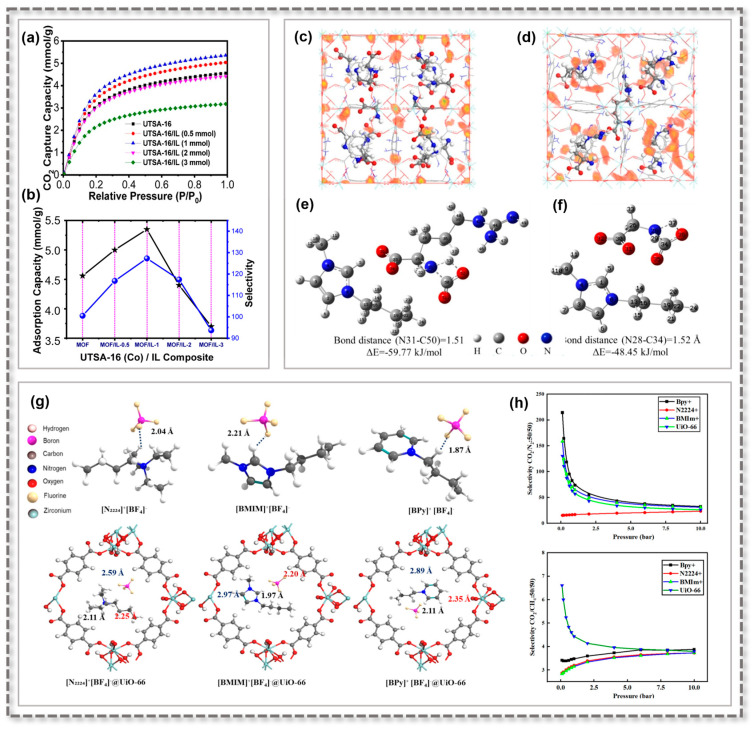
(**a**) Effect of [Bmim][BF_4_] loadings on the CO_2_ adsorption capacity of UTSA-16(Co) at 298 K. (**b**) Modulation of CO_2_/N_2_ selectivity in UTSA-16 induced by IL incorporation (black line: adsorption capacity; blue line: selectivity) [240]. (**c**,**d**) Active sites of CO_2_ within the pores of (**c**) [Bmim][Arg]-NH_2_-UiO-66 and (**d**) [Bmim][Gly]-NH_2_-UiO-66. (**e**,**f**) Binding energies and interaction sites of CO_2_ with (**e**) [Bmim][Arg] and (**f**) [Bmim][Gly] [138]. (**g**) Confined ion-pair configurations and characteristic distances in IL@UiO-66 hybrid materials. (**h**) Pressure-dependent adsorption selectivity of different IL@UiO-66 hybrid materials for CO_2_/N_2_ (top) and CO_2_/CH_4_ (bottom) gas mixtures [189].

Gaikwad et al. incorporated [Bmim][BF_4_] into UTSA-16(Co) via a microwave-assisted solvothermal method. A moderate IL loading of 1 mmol markedly improves CO_2_/N_2_ selectivity and enhances CO_2_ uptake (Figure 17a) [240]. However, higher loadings of 2 mmol or more result in pronounced pore filling, which partially blocks the framework, reduces the BET surface area from 792 to 216 m^2^/g, and decreases the pore volume by approximately 62%, ultimately lowering the CO_2_ adsorption capacity (Figure 17b). By comparison, Zhao et al. demonstrated that the chemical reactivity of ILs, when combined with the ultramicropores of MOFs, effectively mitigates pore blockage [138]. [Bmim]Gly and [Bmim]Arg were impregnated into NH_2_-UiO-66(Zr) and NH_2_-MIL-125(Ti) at loadings of 10–70 wt%. At 50 wt%, the amino-acid-based ILs preserved pore accessibility while generating abundant ultramicropores below 0.65 nm that closely matched the kinetic diameter of CO_2_ (Figure 17c,d). These confined pores strengthen physical adsorption, while the -NH_2_ groups in Gly^−^ and Arg^−^ reversibly form carbamates, producing a synergistic effect of chemical adsorption and confinement (Figure 17e,f). The guanidinium-containing Arg^−^, with its higher basicity and greater number of active sites, enables a CO_2_ uptake of 2.93 mmol/g at 0.0005 bar, along with excellent CO_2_/N_2_ selectivity.

Beyond anion effects, the structure of IL cations also plays a crucial role in determining CO_2_ separation performance. Kumar et al. investigated the influence of different IL cations, imidazolium, pyridinium, and quaternary ammonium, incorporated into UiO-66 with BF_4_^−^ anion, using DFT and grand canonical Monte Carlo (GCMC) simulations [189]. Their results reveal that IL confined within UiO-66 induces ion-pair rearrangement while largely preserving the MOF (Figure 17g). Aromatic cations enhance CO_2_ adsorption through stronger interfacial and π–π interactions. Aliphatic quaternary ammonium cations exhibit lower selectivity due to steric hindrance (Figure 17h). This trend reflects the importance of both IL anions and cations as key parameters governing interfacial interactions and CO_2_ separation performance in IL/MOF hybrid adsorbents, providing important guidance for the rational molecular design of advanced CO_2_ capture materials.

**Figure 18:**
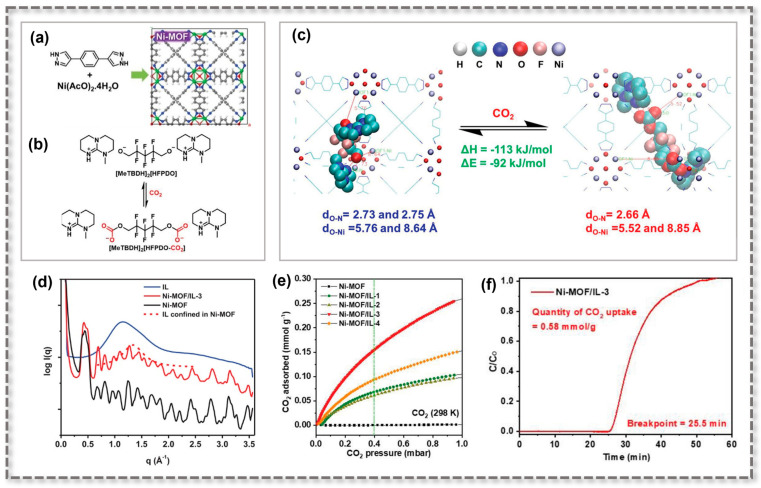
(**a**) Synthetic route of the superbasic [MeTBDH]_2_[HFPDO]. (**b**) Reaction mechanism for carbonate formation via O-C bond formation between the anion and CO_2._ (**c**) DFT-optimized configuration of the SIL confined within the Ni-MOF pore and corresponding reaction energy with CO_2._ (**d**) Small-angle X-ray scattering patterns of pristine SIL, Ni-MOF, and Ni-MOF/IL-3. (**e**) CO_2_ adsorption isotherms of Ni-MOF and Ni-MOF/IL hybrid materials at 298 K. (**f**) Breakthrough curve of Ni-MOF/IL-3 in a 400 ppm CO_2_/He stream at 298 K [127].

Qiu et al. further demonstrated that integrating strong basics with highly stable MOFs can overcome key limitations in DAC [127]. In their work, a SIL, [MeTBDH]_2_[HFPDO], was incorporated into a Ni-based MOF that remains structurally stable under strongly basic and high-ionic-strength conditions (Figure 18a). The deprotonated alkoxide sites of the [HFPDO]^2−^ anion react with CO_2_ to form carbonate adducts, providing a strong chemisorption pathway (Figure 18b). Meanwhile, the SIL is uniformly confined within the MOF nanocavities, increasing the reaction ΔH from −87.8 to −113 kJ/mol, indicating that the confined environment significantly strengthens IL–CO_2_ interactions (Figure 18c,d) [40]. Consequently, the SIL-doped Ni-MOF exhibits markedly enhanced CO_2_ uptake in the low-pressure region, particularly at 0.4 mbar (Figure 18e). The Ni-MOF/IL-3 exhibits a CO_2_ uptake of 0.58 mmol/g at 400 ppm CO_2_ in fixed-bed experiments, more than 14 times higher than that of the pristine MOF (Figure 18f). In addition, the adsorbent displays minute-scale adsorption–desorption kinetics, reversible regeneration at a relatively low temperature at 353 K, and excellent cycling stability, highlighting the great potential of confinement-enhanced chemisorption for DAC applications.

**Figure 19:**
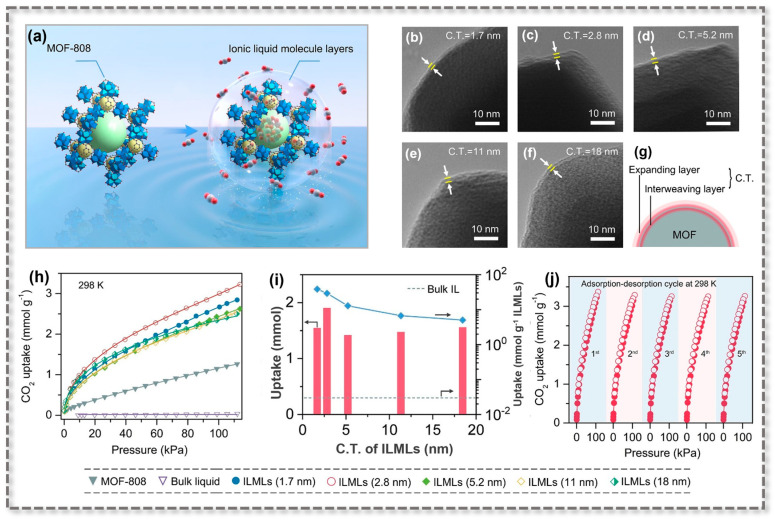
(**a**) Schematic illustration of the solution-assisted assembly of an IL molecular layer on the surface of MOF-808. (**b**–**f**) Transmission electron microscopy (TEM) images of MOF-808 coated with IL molecular layers of varying thicknesses. (**g**) High-resolution TEM image of MOF-808 coated with an 18 nm thick IL molecular layer. (**h**) CO_2_ adsorption isotherms of MOF-808 modified with IL molecular layers of different thicknesses at 298 K. (**i**) Contribution of the IL molecular layer to the CO_2_ adsorption capacity of the composite and comparison of normalized capacities. (**j**) Five-cycle CO_2_ adsorption–desorption performance of MOF-808 modified with a 2.8 nm thick IL molecular layer [241].

In addition, distinct from conventional encapsulation or pore-filling strategies, Zhao et al. proposed an ingenious wrapping–coupling strategy to circumvent pore blockage (Figure 19a) [241]. Instead of introducing the [Bmim][TFSI] into the internal cavities of MOF-808, the [Bmim][TFSI] was impregnated onto the external surface of MOF-808 from a methanol solution, forming a molecular IL layer with a precisely tunable thickness, as an “IL gate”. The outer IL layer enriches CO_2_ through Lewis acid–base and van der Waals interactions, while the intact internal cage-like pore structure of MOF-808 serves as the primary CO_2_ storage space. These components are tightly coupled through a mildly interpenetrated interfacial layer (Figure 19b–g). Notably, when the IL layer thickness is approximately 2.8 nm, the composite achieves a high CO_2_ uptake of 3.00 mmol/g at ambient temperature (Figure 19h). This remarkable enhancement arises from the dramatically increased CO_2_ capacity of the confined IL, which is more than three orders of magnitude higher than that of bulk IL, specifically over 1000-fold (Figure 19i). This effectively overcomes the mass-transfer limitations typically associated with gas–liquid interfaces in conventional IL. Moreover, the hybrid system enables complete CO_2_ desorption at room temperature, allowing nearly zero-energy regeneration during cyclic operation (Figure 19j).

### 4.4. IL/COF Hybrid Materials

COFs are crystalline, porous organic materials constructed from light elements (e.g., C, H, O, N, and B) linked by strong covalent bonds such as B-O, C-N, and C=N [242]. Since the first report of COF materials by Yaghi’s group in 2005, this class of materials has experienced rapid development over the past two decades [243]. In terms of performance, COFs exhibit structural tunability, facile surface functionalization, large specific surface area, ordered pore structures, and high porosity, features that are comparable to those of MOFs [244,245,246]. However, owing to their covalently bonded frameworks, COFs generally display superior thermal and chemical stability compared with MOFs [247,248]. For example, TpPa-1 COF can function as a hydrophobic selective sieve, reducing interference caused by the preferential adsorption of water on CO_2_-affinitive sites [249]. This excellent stability confers clear advantages on COFs for recyclability and long-term applications. Nevertheless, the absence of metal active sites and the relatively large average pore size of COFs may limit their gas capture capacity and separation performance. Despite these limitations, the robust framework structures of COFs, together with their abundant reactive organic functional groups and structural building units, offer versatile opportunities for the incorporation and immobilization of ILs, thereby expanding the possibilities for the construction of IL/COF materials [250,251].

**Figure 20:**
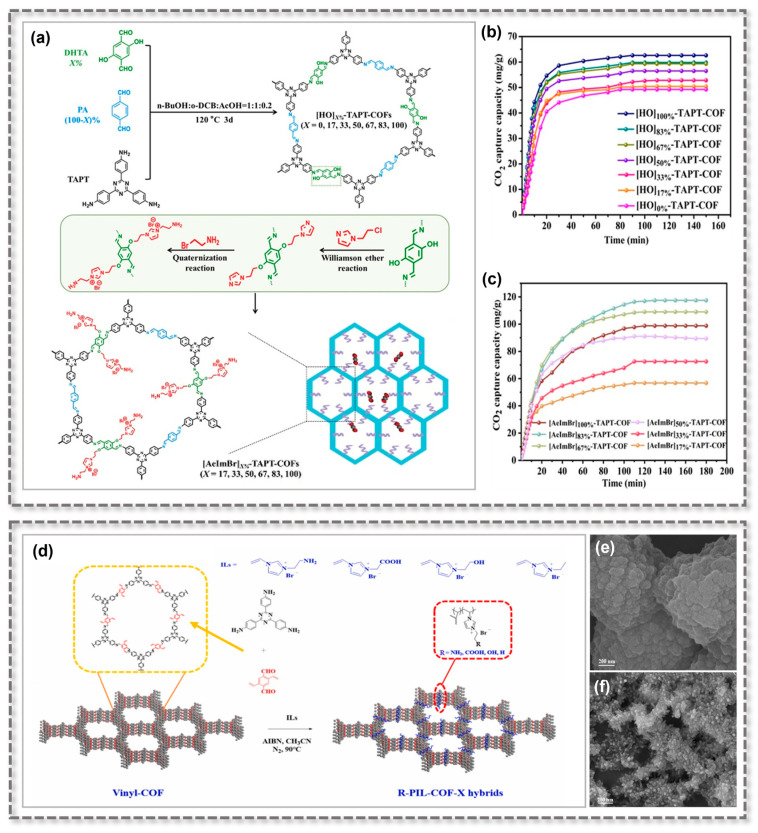
(**a**) Schematic illustration of the synthesis of COFs modified with amino-functionalized ILs via chemical grafting. (**b**) CO_2_ equilibrium adsorption capacities of [HO]_X%_-TAPT-COFs at 298 K and 1 bar. (**c**) CO_2_ adsorption capacities of [AeImBr]_X%_-TAPT-COFs at 298 K and 1 bar [190]. (**d**) Schematic illustration of the synthesis of PIL-COF hybrid materials via copolymerization of vinylene(vinyl)-COF with functionalized imidazolium ILs. (**e**,**f**) Scanning electron microscopy (SEM) image of (**e**) vinyl-COF, (**f**) CH_2_NH_2_-PIL-COF-45% [144].

For example, Yin et al. adopted a post-modification strategy to regulate the density of phenolic hydroxyl groups on the COF pore walls, thereby enabling controllable grafting of ILs within the channels (Figure 20a) [190]. TAPT was condensed with DHPA and TA at different ratios to produce a series of [HO]_X%_-TAPT-COFs with tunable phenolic hydroxyl densities. The IL precursor [AeEIm][Br], containing a bromoethyl group, was subsequently anchored to pore-wall -OH sites via Williamson etherification and further quaternized to form an amino-functionalized imidazolium IL. By varying the -OH content, the spatial density and distribution of IL species within the pores could be precisely regulated. As the phenolic hydroxyl content in [HO]_X%_-TAPT-COFs increased from 0% to 100%, the CO_2_ adsorption capacity rose from 49.2 to 62.6 mg/g, mainly due to the increased specific surface area and the affinity of triazine units for CO_2_ (Figure 20b). After NH_2_-IL grafting, the CO_2_ uptake of [AeImBr]_X%_-TAPT-COFs increased further but exhibited a volcano-type trend. For instance, the CO_2_ adsorption capacity of [AeImBr]_83%_-TAPT-COF increased from 62.6 mg/g for the precursor to 117.4 mg/g (Figure 20c). Under the conditions tested, moderate IL functionalization introduces additional active sites while maintaining pore accessibility, whereas excessive grafting blocks the channels and reduces adsorption performance.

Compared with point-like grafting on pore walls, Chen et al. introduced polymerization sites into the COF, transforming IL immobilization from discrete grafting into chain-like structures and enabling molecular-scale crosslinking between the COF and PILs [144]. A vinyl-containing DVTP monomer was incorporated during COF synthesis to distribute polymerizable double bonds along the pore walls uniformly (Figure 20d). Subsequent in situ free-radical polymerization of vinyl-imidazolium IL monomers generated an interpenetrating polymeric IL network within the channels, forming a “ship-in-a-bottle” architecture. This design creates hierarchical micro–meso–macroporous structures while chemically integrating polymeric IL chains with the COF (Figure 20e,f), thereby facilitating CO_2_ diffusion and adsorption. Owing to the strong polarity of amino-functionalized IL monomers, CH_2_NH_2_-PIL-COF-45% achieves a CO_2_ uptake of 59.5 mg/g at 1 bar.

**Figure 21:**
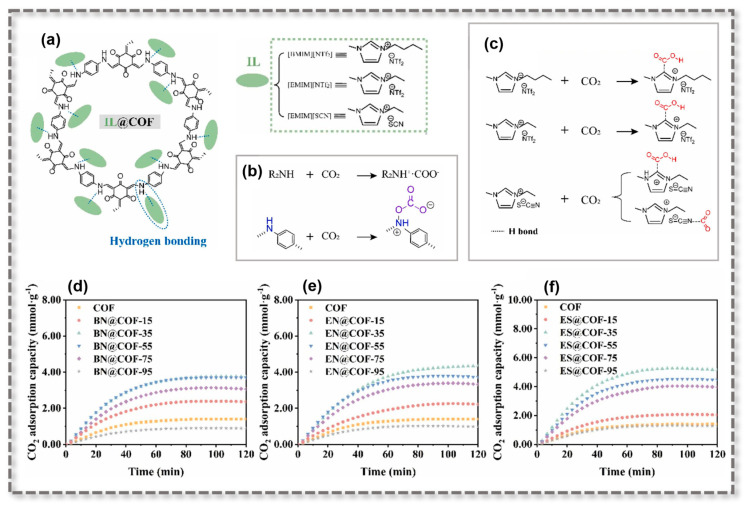
(**a**) Schematic of IL@COF-X hybrid materials prepared by impregnating COFs with three ILs. (**b**,**c**) Synergistic CO_2_ adsorption mechanisms of COFs and ILs. (**d**–**f**) CO_2_ uptake vs. IL loading from 15 to 95 wt% at 333 K and 1 bar under simulated flue gas of 15% CO_2_/85% N_2_ for (**d**) [Bmim][NTf_2_], (**e**) [Emim][NTf_2_], and (**f**) [Emim][SCN]-modified COFs [252].

Beyond covalent modification strategies, Zheng et al. introduced an IL@COF coupling mode based on non-covalent confined and hydrogen-bond networks, expanding IL immobilization mechanisms in COF channels [252]. Specifically, ILs with multiple hydrogen-bond donors or acceptors, including [Bmim][NTf_2_], [Emim][NTf_2_], and [Emim][SCN], were incorporated into TpPa-1 COFs featuring uniform pores and polar functional groups via post-synthetic impregnation (Figure 21a). The ILs form stable hydrogen bonds with pore-wall C=O and C-N sites, enabling effective confinement without covalent bonding while preserving crystallinity and pore connectivity. This hydrogen-bond-mediated environment enhances CO_2_ adsorption potential compared with physisorption and promotes carbamate formation by activating lone-pair-rich nitrogen atoms as nucleophilic sites (Figure 21b). CO_2_ affinity is largely dictated by the anion, whereas the cation indirectly influences adsorption via viscosity, molecular size, and pore accessibility (Figure 21c) [253,254]. At low loadings of 15 wt%, ILs only partially penetrate pores, with higher viscosity [Bmim][NTf_2_] mainly on the surface, providing initial adsorption sites, yielding higher uptake for BN@COF-15 (Figure 21d–f). At higher loadings of ≥35 wt%, pore filling increases diffusion resistance, and lower viscosity [Emim][NTf_2_] exhibits superior kinetics. Theoretical calculations indicate that [SCN]^−^ binds CO_2_ more strongly than [NTf_2_]^−^, displaying pronounced chemisorption behavior. Overall, ES@COF-35 achieves the highest CO_2_ adsorption capacity and selectivity in this series.

**Figure 22:**
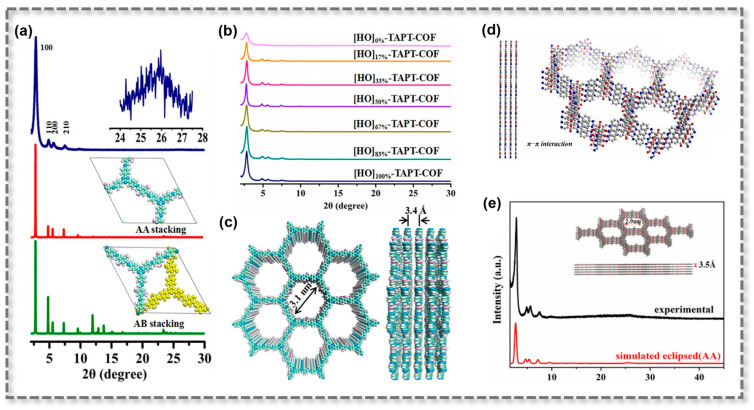
(**a**) Experimental powder X-ray diffraction (PXRD) pattern of [HO]_100%_-TAPT-COF compared with simulated AA and AB stacking models. (**b**) PXRD patterns of [HO]_X%_-TAPT-COFs with varying phenolic hydroxyl contents. (**c**) Expanded AA-stacked unit-cell model of [HO]_100%_-TAPT-COF [190]. (**d**) Expanded AA-stacked unit-cell model of TpPa-1 COF [252]. (**e**) Experimental PXRD pattern of vinyl-COF (black) compared with the simulated AA stacking model (red) [144].

Notably, the COF employed in the IL-introduction strategies described above predominantly adopts a two-dimensional AA-stacked architecture (Figure 22), the typical structural motif of many 2D COFs. This configuration aligns adjacent layers to generate ordered one-dimensional channels, providing an ideal platform for the confined immobilization and functional integration of ILs [255,256,257]. The resulting nanopores spatially regulate the distribution of ILs and suppress aggregation and leaching. At the same time, tunable pore-wall functionalities such as -OH, -NH_2_, and vinyl groups enable chemical grafting, hydrogen bond confinement, or in situ polymerization [258]. Such structural features not only maintain high porosity but also enhance CO_2_ affinity through the synergistic coupling of confinement and interaction. Nevertheless, post-synthetic IL incorporation may perturb the COF and compromise structural stability [259,260]. Therefore, developing facile construction strategies that balance framework integrity and CO_2_ capture performance is essential to advance practical IL–COF hybrid materials.

After systematically discussing the design strategies and CO_2_ capture performance of IL hybrid systems based on silica, carbon materials, MOFs, and COFs, it becomes evident that different porous supports possess distinct advantages and limitations with respect to confinement effects, interfacial interactions, mass-transfer behavior, and structural stability. To more clearly compare the structural features and performance differences among these material systems, a systematic comparative summary is provided in Table 5.

## 5. Life-Cycle Assessment and Techno-Economic Analysis

IL hybrid porous materials show considerable promise for CO_2_ capture. However, their practical deployment depends not only on intrinsic material performance but also on life-cycle sustainability and system-level economic feasibility [261,262]. The high cost of IL synthesis, the complexity of hybrid material fabrication, and limited long-term stability may offset their performance advantages when evaluated across the entire life cycle [263,264]. Therefore, a comprehensive assessment of their sustainability at the system level is essential.

### 5.1. Life-Cycle Environmental Impacts

**Figure 23:**
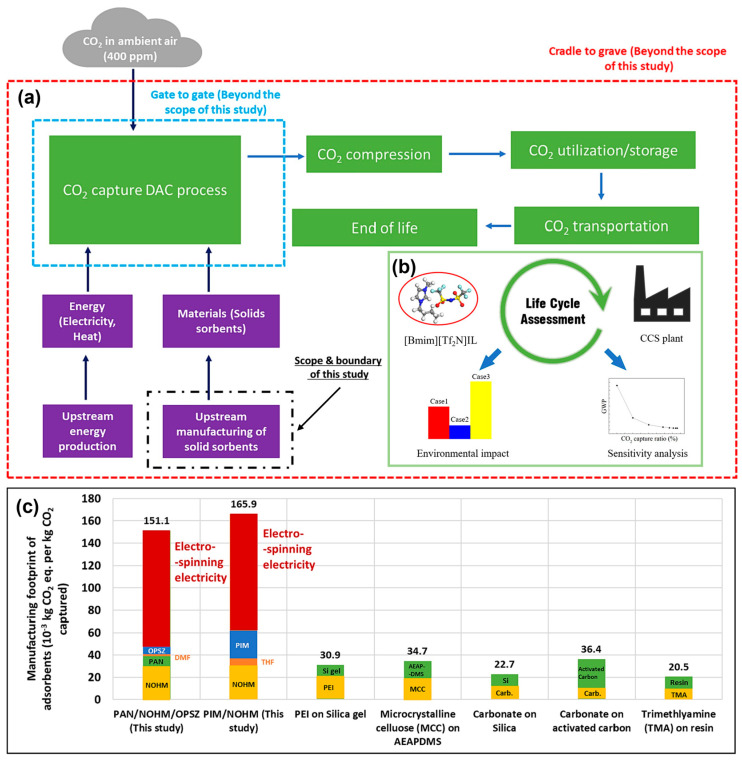
(**a**) System boundary of the LCA for fiber-encapsulated nanoscale hybrid materials used in DAC [271]. (**b**) LCA framework for [Bmim][NTf_2_]-based CCS processes [263]. (**c**) Comparative GWP of various porous sorbents for DAC [271].

During raw material acquisition and synthesis, the green preparation of s represents a major bottleneck in their life-cycle assessment (LCA) [265,266,267]. A LCA of the carbon capture and storage process based on [Bmim][NTf_2_] conducted by Zhang showed that solvent production dominates the overall environmental impact, contributing about 90% (Figure 23b) [263]. Specifically, producing 1 kg of [Bmim][NTf_2_] results in a global warming potential (GWP) of 15.0 kg CO_2_ eq. In contrast, the impact during the use stage for capturing 1 kg CO_2_ is less than one-thousandth of this value, underscoring the dominant influence of upstream synthesis. Further analysis indicates that anion synthesis is the principal environmental hotspot. For example, the production of the anion precursor LiNTf_2_ imposes substantially higher burdens in GWP, human toxicity potential (HTP), and acidification potential (AP) than the cation precursor [Bmim][Cl], mainly due to its complex synthesis route, high energy demand, and significant chemical losses [268]. In addition, most functionalized ILs rely on petrochemical routes involving high-purity halogenated hydrocarbons, strong bases, and noble-metal catalysts, leading to high energy consumption and emissions [269,270]. Consistently, life-cycle studies of materials used in DAC show that the manufacturing stage often contributes more to GWP than the operational stage, further highlighting the decisive role of upstream synthesis in determining the environmental performance of ILs (Figure 23a,c) [271].

In addition, the coupling of ILs with porous materials may increase the overall environmental burden. Common strategies, including impregnation, in situ growth, and covalent grafting, typically require organic solvents and must be performed under strictly anhydrous conditions [272,273]. Meanwhile, the high viscosity of ILs can lead to non-uniform distribution within nanopores, causing pore blockage or aggregation that reduces the effective surface area and restricts CO_2_ diffusion. These structural constraints not only weaken adsorption performance but may also increase operational energy demand. More importantly, under realistic flue gas conditions, components such as water vapor and oxygen may induce IL degradation or damage the porous materials, resulting in noticeable capacity loss after repeated adsorption–desorption cycles [274,275,276]. Therefore, from an LCA perspective, material stability and regenerability are key factors governing overall environmental performance.

At the end of the life stage, the environmental persistence of ILs also warrants attention. Some conventional ILs, particularly those containing fluorinated anions, exhibit low biodegradability in natural environments and may pose ecological risks if not effectively recovered [277,278]. Although biodegradable ILs derived from precursors such as lactic acid or glycerol have been developed in recent years, their CO_2_ capture performance still falls short of industrial requirements [278,279,280]. Therefore, establishing efficient recovery and recycling strategies for ILs, such as supercritical CO_2_ extraction or solvent-based separation, represents an important approach to reducing life-cycle environmental impacts.

### 5.2. Economic and Industrialization Challenges

Beyond environmental considerations, economic feasibility remains a key constraint on the scale-up of IL/porous hybrid materials. Functionalized ILs typically cost 100–500 USD/kg, far exceeding conventional amine solvents such as 30 wt% MEA solutions, which cost about 1–2 USD/kg [10,109]. Even with hybridization strategies that reduce IL loading, a mass fraction above 10 wt% in the composite can still substantially increase overall material costs [281,282,283]. Meanwhile, porous materials such as high-performance MOFs and COFs also face high synthesis costs [284,285]. MOFs rely on expensive metal salts and organic linkers, and challenges in metal sourcing and hydrothermal stability further limit their scalability [286]. Although mesoporous silica and carbon materials are cheaper, their physisorption-dominated mechanisms result in low capacity under low partial pressures, often requiring larger adsorption beds or more frequent cycling, thereby increasing energy consumption [264,287]. To provide a clearer comparison of the economic burden associated with different porous supports, the synthesis costs of representative porous materials are summarized in Table 6.

Regeneration energy and mass transfer limitations further impact system-level economics [294]. The high viscosity of ILs within porous matrices restricts CO_2_ diffusion, extending adsorption–desorption cycles and resulting in actual regeneration energy demands exceeding 2.5 GJ/t CO_2_ [295,296,297]. Structural degradation over repeated cycling increases adsorbent replacement frequency, driving operational costs higher. Techno-economic analyses (TEAs) indicate that CO_2_ capture using IL hybrid materials typically costs 80–200 USD/t CO_2_, well above the commercially viable target of 30–50 USD/t CO_2_ (Table 7) [298]. Even in hybrid liquefaction and low-temperature adsorption systems optimized with waste heat, costs rarely fall below 60 USD/t CO_2_, with the high expense primarily arising from elevated material prices, substantial regeneration energy requirements, and increased equipment investment due to system complexity [299,300].

Beyond economic barriers, long-term stability under realistic operating conditions remains a critical yet insufficiently explored challenge. In industrial applications, IL-based hybrid adsorbents must function in complex and often harsh environments [308,309]. Although many ILs possess good intrinsic thermal stability, they may still undergo degradation or leakage from porous supports at elevated temperatures [310]. Confinement within nanoscale pores can further modify their decomposition pathways, and in some cases, strong interactions with pore walls or catalytic effects from the support may even accelerate degradation [311]. Industrial flue gases typically contain corrosive species such as H_2_S, SO_2_, NO_x_, and water vapor [312]. These impurities can react with ILs or degrade porous supports. For example, SO_2_ may strongly interact with certain ILs, inducing structural changes that reduce CO_2_ uptake capacity and regeneration efficiency [308,312]. Consequently, the long-term stability of IL-based hybrid materials in such chemically complex environments remains poorly understood.

During gas separation, adsorbents are also exposed to mechanical stresses from gas flow and pressure fluctuations associated with adsorption–desorption cycling [313]. Accordingly, mechanical strength and abrasion resistance are essential for reliable long-term operation. However, systematic studies on the mechanical durability of IL-based hybrid adsorbents under cyclic conditions remain limited [314,315]. Taken together with economic constraints, these stability issues underscore the need for more comprehensive investigations that extend beyond intrinsic material properties to include durability, recyclability, and system-level reliability. Without such efforts, the translation of laboratory-scale advances into industrial applications will remain significantly constrained.

### 5.3. Sustainability Strategies

**Figure 24:**
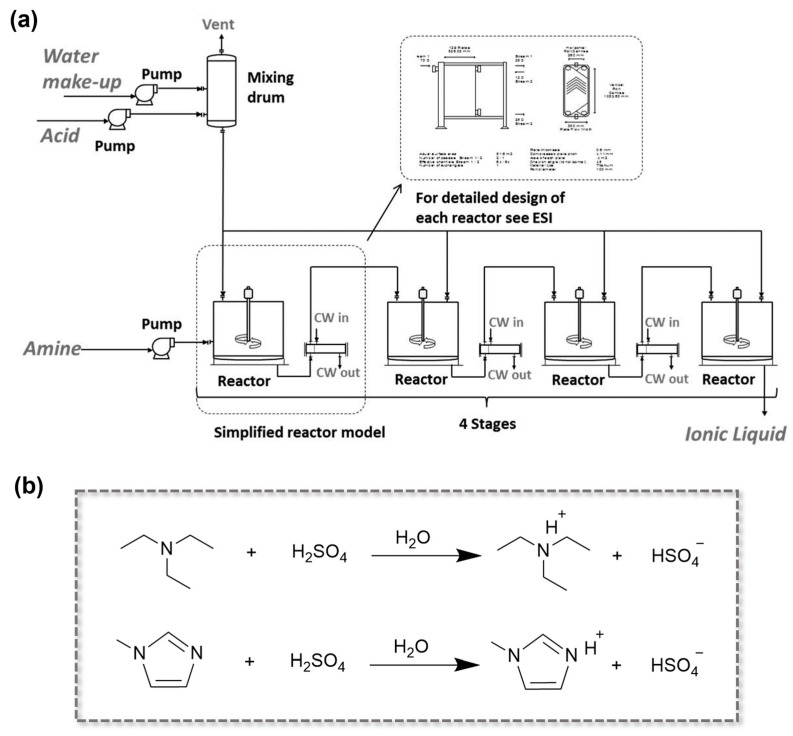
(**a**) Schematic diagram of the IL-enhanced synthesis process [311]. (**b**) Chemical structures and synthesis reactions of two protic ILs [HNEt_3_][HSO_4_] and [C_1_Him][HSO_4_].

Despite ongoing challenges in life-cycle performance and economic feasibility, the highly tunable structures of IL/porous hybrid materials offer significant opportunities to optimize key parameters, including CO_2_ selectivity, absorption capacity, and regeneration energy consumption. Developing low-cost, green synthetic routes for ILs is a critical step toward reducing both overall costs and environmental footprint [316,317,318]. For example, the synthesis of the protic IL [HNEt_3_][HSO_4_] from basic raw materials requires substantially fewer steps than that of commonly used aprotic dialkylimidazolium ILs (e.g., [Bmim][BF_4_]), which typically involve more synthetic steps [319,320]. This marked simplification, as reported by Chen et al., reduces the estimated production cost to approximately 1.24 USD/kg, comparable to that of bulk organic solvents such as ethyl acetate or acetone (Figure 24). These results indicate that the high cost of ILs is not an intrinsic feature of the concept itself but rather arises from the design of structurally complex molecules. Similar advantages have been also demonstrated in separation processes. Meindersma et al. showed that using specific ILs to extract aromatics from naphtha cracking feedstocks reduces process energy consumption to 32 MW, well below the 160 MW required by conventional cyclodextrin-based processes, while generating annual revenues of up to €48 million per unit [321].

The development of phase-change or low-viscosity ILs can further improve mass transfer and reduce pumping energy. For example, Zheng et al. developed a reversible-polarity IL, [IPDAH][Im], which undergoes a solid–liquid phase transition upon CO_2_ absorption, achieving a thermal desorption energy of only 1.23 GJ/t CO_2_, roughly 67% lower than the MEA system [322]. Additionally, ILs derived from choline, amino acids, or other biomass-based precursors not only reduce dependence on petrochemical feedstocks but also have the potential to lower carbon emissions during synthesis [323,324,325].

For porous organic frameworks, high synthesis costs are largely attributable to conventional solvothermal methods, which require elevated temperatures and large volumes of organic solvents such as DMF, resulting in material prices of 35–71 USD/kg [326,327]. Mason et al. demonstrated that combining liquid-assisted grinding with aqueous-phase synthesis, alongside optimized reaction conditions and improved yields, can reduce MOF production costs to approximately 10 USD/kg [328]. Furthermore, replacing portions of expensive frameworks with low-cost, structurally robust porous supports, such as biomass-derived carbons or industrial byproducts, offers an effective route to reduce adsorbent costs [329]. Coupling CO_2_ capture with subsequent catalytic or electrochemical conversion, such as capture-enabled electrochemical CO_2_ reduction, to construct integrated capture-conversion systems further enables partial cost offset through the generation of high-value chemicals [11,330,331,332]. Collectively, these results indicate that the high costs of both ILs and porous materials are not intrinsic material properties but are strongly governed by synthetic routes, processing strategies, and scale-up level.

Overall, IL/porous hybrid materials represent a highly designable class of advanced materials for CO_2_ capture. However, their future development should extend beyond improvements in adsorption performance and instead emphasize stronger integration among material design, process optimization, and system-level engineering. From an LCA perspective, optimizing feedstock sources, synthetic pathways, and material recycling strategies will be crucial for advancing these materials toward industrially viable carbon capture technologies.

## 6. Conclusions and Outlook

ILs/porous hybrid materials have evolved beyond simple immobilization systems into confinement-enhanced adsorption platforms, in which nanoscale confinement fundamentally alters the physicochemical behavior of ILs. Through the synergistic regulation of free volume, interfacial interactions, ion arrangement, active-site accessibility, and adsorption thermodynamics, the hybrid systems effectively overcome the intrinsic limitations of bulk ILs, including high viscosity, slow gas diffusion, and severe mass-transfer resistance. As a result, ILs/porous hybrid materials exhibit significantly enhanced CO_2_ capture capacity, selectivity, regeneration efficiency, and cyclic stability, demonstrating broad potential for applications ranging from post-combustion carbon capture to DAC.

Despite substantial progress in this field, several challenges and knowledge gaps remain and require further investigation. First, the fundamental mechanisms governing confinement-induced IL restructuring and interfacial adsorption behavior remain insufficiently understood, particularly under realistic multicomponent and humid conditions. Advanced in situ characterization techniques, molecular dynamics, and multiscale theoretical models are still required to establish quantitative relationships among pore structure, IL organization, transport behavior, and adsorption thermodynamics. Second, excessive IL loading may cause pore blockage, diffusion resistance, and active-site shielding, indicating that achieving an optimal balance among IL loading, pore accessibility, and confinement-enhanced effects remains a central challenge. Third, the long-term stability and lifetime of hybrid materials under cyclic operation and complex conditions, especially regarding IL leakage, structural degradation, and moisture tolerance, still need to be further improved for industrial implementation. In addition, challenges also persist in the precise construction, scalable production, and cost-effective synthesis of IL hybrid systems. Furthermore, comprehensive assessments of energy consumption, carbon footprint, and economic feasibility across the entire process chain are still needed.

**Figure 25:**
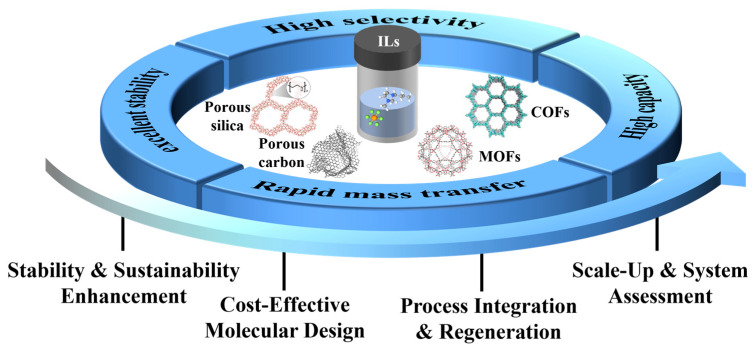
Advantages and future perspectives of IL-hybridized porous materials for carbon capture.

Future research should move beyond simply maximizing CO_2_ uptake toward the rational design of confinement-enhanced IL hybrid systems with balanced adsorption capacity, selectivity, kinetics, stability, and regeneration energy consumption. Research on ILs/porous hybrid materials for carbon capture is expected to focus on the following directions (Figure 25):

(1) Molecular design and synthesis of low-cost, high-performance ILs. Breaking the cost barrier requires the development of alternative feedstocks and optimized synthetic routes, such as employing biomass-derived components or industrial by-products as precursors and developing one-step or solid-state synthesis methods. Meanwhile, functionalized ILs with both high CO_2_ affinity and low viscosity should be designed to balance improved thermodynamic adsorption performance with efficient mass-transfer kinetics.
(2) Rational regulation of confinement-enhanced hybrid architectures. Future studies should focus on constructing hierarchical pore structures and precisely regulating the interfacial microenvironment of confined ILs to optimize free volume, ion arrangement, active-site accessibility, and mass-transfer pathways. Moreover, advances in in situ regeneration of ILs and structural repair of porous materials are needed to enhance the recyclability and economic viability of hybrid materials. The integration of hybrid adsorbents with catalytic conversion, electrochemical systems, or other processes should also be explored to construct low-energy and highly efficient carbon capture platforms.
(3) Stability enhancement and environmental compatibility of hybrid adsorbents. For practical conditions such as flue gas treatment and DAC, hydrophobic ILs with strong resistance to moisture and impurity gases should be developed. In parallel, following the principles of green chemistry, comprehensive evaluation frameworks covering the entire life cycle, from material synthesis and operation to disposal, should be established to ensure environmental sustainability.
(4) AI-driven material discovery and system-level optimization for carbon capture. Future research should transition from conventional data-driven approaches toward AI-driven frameworks for the rapid discovery of confinement-enhanced ILs/porous hybrid materials. By integrating high-throughput simulations, experimental data, and machine learning, these platforms can enable the inverse design of IL structures and porous architectures for targeted CO_2_ capture. They may also clarify structure–property relationships governing adsorption thermodynamics, mass transfer, confinement effects, and stability. Coupling AI-assisted material design with process modeling can further guide the development of optimized DAC systems, supporting the co-design of materials and processes for improved efficiency, lower energy consumption, and scalable industrial applications.

Overall, the transition from bulk ILs to confinement-enhanced IL hybrid adsorbents provides a promising pathway toward efficient, stable, and energy-efficient CO_2_ capture technologies. With continued advances in confinement engineering, interfacial regulation, multiscale material design, and AI-assisted design, IL-based hybrid systems are expected to play an increasingly important role in future CCUS technologies and global carbon-neutral energy systems.

## Figures and Tables

**Table 2:** Hybridization Strategies with Advantages and Limitations.

Strategy Category		Advantage	Limitation	Ref.
In situ construction	Solvothermal synthesis	Framework–IL co-assembly Strong interfacial integration High structural synergy	Limited IL stability Stringent reaction conditions	[142,153]
	Ionothermal synthesis	Solvent-free Structure-directing role of IL	Size-matching constraints Potential pore blockage	[143,151,152,165,166,167]
	In situ polymerization	Strong IL immobilization Formation of stable poly(IL) networks	Complex polymerization kinetics Pore control requirements	[154]
Post-synthetic modification	Physical impregnation	Simple and versatile Mild conditions Preserves IL structure	Weak interactions and IL leaching Non-uniform distribution Pore blockage Reliance on organic solvents	[145,155,156]
	Capillary filling	Uniform IL distribution Solvent-free Environmentally friendly	Predominantly physically confined Restricted pore size requirements Confinement-induced IL alteration	[146,157,158,159]
	“Ship-in-a-bottle” encapsulation	Effective confined Minimized IL leakage Controlled nanospaces	Stringent reaction conditions Diffusion limitations Risk of pore blockage	[147,160]
	Chemical grafting	Strong covalent attachment High stability Tunable interfaces	Multistep synthesis Possible pore structure alteration Site-limited loading	[144,163]

**Table 3:** Advantages and limitations of porous materials for carbon capture.

Material Type	Advantage	Limitation	Ref.
Porous silica materials	High surface area Tunable porosity Facile functionalization Good mechanical stability	Weak CO_2_ affinity Limited hydrothermal stability Structural degradation	[22,173,174,175]
Porous carbon materials	Low cost and scalability Humidity tolerance Tunable porosity High stability	Low polarity and CO_2_ affinity Poor low-pressure selectivity Pressure-dependent capacity	[176,177,178]
MOFs	Ultrahigh surface area Structurally tunable Structural tunability Strong CO_2_ affinity/selectivity Effective at low pressure	High cost Moisture sensitivity Limited durability Metal source constraints	[46,179,180,181,182]
COFs	Ordered channels Metal-free High stability Tunable structure Low density	Large pore size Weak binding sites Poor low-pressure performance Long synthesis time	[183,184,185]

**Table 4:** Effect of IL loading of ZIF-8 on CO_2_ adsorption performance.

No.	Material	Loading (%)	P_CO2_ (bar)	T (K)	Surface Area (m^2^/g)	Pore Volume (cm^3^/g)	CO_2_ Uptake (mmol/g)	αCO_2_/N_2_	Ref.
1	ZIF-8	0	10	313	~1650	~0.70	~5	~7	[236]
2	ZIF-8	0	1	313	~1650	~0.70	~0.4	~5	[236]
3	ZIF-8	0	0.1	313	1649 ± 20	0.70	~0.03	~3	[237]
4	[Emim][Gly]@ZIF-8	10	0.1	313	1318 ± 9.6	0.54	~0.06	~1	[237]
5	[Emim][Gly]@ZIF-8	20	0.1	313	887 ± 7.7	0.34	~0.2	~10	[237]
6	[Emim][Gly]@ZIF-8	30	0.1	313	634 ± 6	0.24	~0.65	~28	[237]
7	[Emim][Ala]@ZIF-8	30	0.1	313	718 ± 6.9	0.26	~0.7	~18	[237]
8	[Emim][Ac]@ZIF-8	30	0.1	313	-	-	~0.6	~5	[124]
9	[Bmim][Ac]@ZIF-8	30	0.1	313	386	0.15	~0.7	~7.5	[124]
10	[Bmim][PF_6_]@ZIF-8	30	0.1	285	415	0.22	-	~24.2	[238]
11	[Bmim][SCN]@ZIF-8	30	0.1	313	60.56	-	-	~20	[239]

αCO_2_/N_2_: The ideal selectivity of CO_2_ over N_2_, calculated as the ratio of Henry’s constants or the initial slopes of the adsorption isotherms of CO_2_ and N_2_.

**Table 5:** Comparative summary of IL-based hybrid adsorbents constructed on different porous materials for CO_2_ capture [40,42,144,187,192,240].

Comparison Aspect	Silica-Based Hybrid Materials	Carbon-Based Hybrid Materials	MOF-Based Hybrid Materials	COF-Based Hybrid Materials
Pore characteristics	Ordered mesoporous channels	Tunable micro-/mesoporous structures	Ultramicroporous cages Tailorable frameworks	Ordered 1D channels with AA stacking
IL confinement mode	Silanol anchoring Physical impregnation	π–π interactions Electronic modulation	Nanocage encapsulation Metal-site coordination	Hydrogen-bond networks Covalent grafting In situ polymerization
Dominant CO_2_ capture mechanism	Functionalized-IL chemisorption	Electronic-regulated physisorption	Physisorption–chemisorption IL-gating effects	Confinement-enhanced chemisorption
Optimal IL loading	Moderate to high	Low to moderate	Moderate to high	Moderate
CO_2_/N_2_ selectivity	Moderate to high	Moderate	High	Moderate to high
Advantages	Low cost Ordered porosity Facile functionalization	Hydrophobicity Moisture tolerance Electrical conductivity	Ultrahigh surface area Tunable frameworks Excellent low-pressure capture performance	High thermal stability High chemical stability Metal-free framework Uniform channels
Limitations	Moderate hydrothermal stability	Broad pore-size distribution Limited selectivity	Moisture sensitivity Relatively high cost	Relatively large pore size Time-consuming synthesis

**Table 6:** Reporting cost estimates for adsorbents.

Adsorbent	Cost of Raw Materials	Transportation Cost	Cost of Required Chemicals	Cost of Energy	Net Cost	Other Costs	Other Costs as % of Net Cost	Total Cost	Ref.
Mesoporous silica (sol–gel method)	N.R.	N.I.	6.51	N.I.	6.51	N.I.	N.I.	6.51	[288]
Mesoporous silica (template method)	N.R.	N.I.	24.03	N.I.	24.03	N.I.	N.I.	24.03	[288]
Graphene oxide	68.75	N.I.	130	N.I.	198.75	N.I.	N.I.	198.75	[289]
Graphene oxide	N.I.	N.I.	N.I.	N.I.	N.I.	N.I.	N.I.	6800	[290]
Magnetite/Non-oxidative graphene composites	N.I.	N.I.	N.I.	N.I.	N.I.	N.I.	N.I.	2000	[291]
Poly(3-aminobenzoic acid/graphene oxide/cobalt ferrite) nanocomposite	N.I.	N.I.	2260	700	2960	N.I.	N.I.	2960	[292]
Zeolitic imidazolate framework-8	N.I.	N.I.	51.8	762.2	814	N.I.	N.I.	814	[293]

N.R. = Not required, N.I. = Not included; All costs are based on 1 kg of adsorbents. All costs have been converted to USD.

**Table 7:** Comparison of life-cycle-related characteristics of different hybrid materials.

Material System	Capture Cost (USD/t CO_2_)	Regeneration Energy (GJ/t CO_2_)	Material Cost (USD/kg ads)	Adsorption Capacity (mmol/g)	Cycling Stability	Ref.
MOF-superbase IL	100–180	2.0–3.0	Very high	1.5–2.5 (400 ppm)	Excellent	[127]
ZIF-8-amine IL	120–220	2.2–3.2	High	2.0–3.0 (1 bar)	Moderate	[301]
Biochar-IL hybrid	90–160	1.8–2.8	Moderate	1.47 (1 bar)	Good	[302]
Fiber-encapsulated IL nanohybrid	150–200	>2.5	High	-	Moderate	[271]
Hybrid liquefaction and low-temperature adsorption systems	80–200	>2.5	Moderate	-	Good	[299]
Zeolite 13X	50–100	0.79–4.5	Low	0.4–0.6 (1 bar)	Excellent	[303,304]
Activated carbon	50–90	2.0–4.5	Low	0.3–0.5 (1 bar)	Excellent	[304]
MEA 30 wt%	50–100	3.5–4.5	Low	1.97 (0.1 bar)	Poor	[305,306,307]

## Data Availability

Data sharing is not applicable to this article as no new data were created or analyzed in this study.

## Nomenclature

13X	Type 13X zeolite
AeImBr	Aminoethyl-imidazolium bromide
AFI	AlPO_4_-5-type framework
CH_4_	Methane
-CHO	Aldehyde
CO_2_	Carbon dioxide
-COOH	Carboxyl
DHPA	2,5-Dihydroxyterephthalaldehyde
DMF	*N, N*-dimethylformamide
DVTP	2,5-Divinylterephthalaldehyde
EDDA	Ethylenediamine-N, N′-diacetate
HIDA	Hydroxy-iminodiacetate
HKUST-1	Hong Kong University of Science and Technology-1
IDA	Iminodiacetate
LiNTf_2_	Lithium bis(trifluoromethanesulfonyl)imide
LTA	Linde type A framework
MAPOs	Metal aluminophosphates
MCM-41	Mobil Composition of Matter No. 41
MCM-48	Mobil Composition of Matter No. 48
MEA	Monoethanolamine
MIL-53	Materials of Institute Lavoisier-53
MOF-74	Also known as CPO-27
N_2_	Nitrogen
Na-Y	Sodium Y zeolite
-NH_2_	Amino
NTA	Nitrilotriacetate
-OAc	Acetate
-OH	Phenolic hydroxyl
OPSZ	Organopolysilazane
PAN/	Polyacrylonitrile
SBA-15	Santa Barbara Amorphous-15
-Si(OCH_2_CH_3_)_3_	Triethoxysilyl
SiO_2_	Silicon dioxide
SOD	Sodalite-type framework
TA	Terephthalaldehyde
TAPT	2,4,6-Tris(4-aminophenyl)-1,3,5-triazine
TpPa-1	1,3,5-Triformylphloroglucinol–p-phenylenediamine
UiO-66	University of Oslo-66
UTSA-16	University of Texas at San Antonio-16
ZIF-8	Zeolitic imidazolate framework-8
[18C6-K][Pro]	Potassium(18-crown-6) prolinate
[4Mbp][BF_4_]	4-Methyl-N-butylpyridinium tetrafluoroborate
[AeEIm][Br]	1-Aminoethyl-3-ethylimidazolium bromide
[AlCl_4_]^−^	Tetrachloroaluminate
[Amim][NTf_2_]	1-Allyl-3-methylimidazolium bis(trifluoromethylsulfonyl)imide
[B(CN)_4_]^−^	Tetracyanoborate
[BF_4_]^−^	Tetrafluoroborate
[Bmim][Ac]	1-Butyl-3-methylimidazolium acetate
[Bmim][Arg]	1-Butyl-3-methylimidazolium argininate
[Bmim][BF_4_]	1-Butyl-3-methylimidazolium tetrafluoroborate
[Bmim][CF_3_SO_3_]	1-Butyl-3-methylimidazolium trifluoromethanesulfonate
[Bmim][Cl]	1-Butyl-3-methylimidazolium chloride
[Bmim][Gly]	1-Butyl-3-methylimidazolium glycinate
[Bmim][LEU]	1-Butyl-3-methylimidazolium leucinate
[Bmim][NO_3_]	1-Butyl-3-methylimidazolium nitrate
[Bmim][NTf_2_]	1-Butyl-3-methylimidazolium bis(trifluoromethanesulfonyl)imide
[Bmim][PF_6_]	1-Butyl-3-methylimidazolium hexafluorophosphate
[Bmim][SCN]	1-Butyl-3-methylimidazolium thiocyanate
[Bmim][TFSI]	1-Butyl-3-methylimidazolium bis(trifluoromethylsulfonyl)imide
[Bmim]^+^	1-Butyl-3-methylimidazolium
[Br]^−^	Bromide
[C_1_Him][HSO_4_]	1-Methylimidazolium hydrogen sulfate
[C_4_TPIm][Cl]	1-Butyl-3-(3-triethoxysilylpropyl)imidazolium chloride
[C_8_H_4_F_13_mim][NTf_2_]	1-(Perfluorooctyl)-3-methylimidazolium bis(trifluoromethylsulfonyl)imide
[Omim][NTf_2_]	1-Octyl-3-methylimidazolium bis(trifluoromethylsulfonyl)imide
[Cl]^−^	Chloride
[CNC_3_mim][NO_3_]	1-(3-Hyanopropyl)-3-methylimidazolium nitrate
[DCA]^−^	Dicyanamide
[Deme][NTf_2_]	N,N-Diethyl-N-methyl-N-(2-methoxyethyl)ammonium bis(trifluoromethylsulfonyl)imide
[Emim][Ac]	1-Ethyl-3-methylimidazolium acetate
[Emim][Ala]	1-Ethyl-3-methylimidazolium alaninate
[Emim][BF_4_]	1-Ethyl-3-methylimidazolium tetrafluoroborate
[Emim][Gly]	1-Ethyl-3-methylimidazolium glycinate
[Emim][NTf_2_]	1-Ethyl-3-methylimidazolium bis(trifluoromethylsulfonyl)imide
[Emim][OAc]	1-Ethyl-3-methylimidazolium acetate
[Emim][SCN]	1-Ethyl-3-methylimidazolium thiocyanate
[Emim][TfO]	1-Ethyl-3-methylimidazolium trifluoromethanesulfonate
[Emim][TFSA]	1-ethyl-3-methylimidazolium bis(trifluoromethylsulfonyl)amide
[Hmim][BF_4_]	1-Hexyl-3-methylimidazolium tetrafluoroborate
[Hmim][NTf_2_]	1-Hexyl-3-methylimidazolium bis(trifluoromethylsulfonyl)imide
[Hmim][PF_6_]	1-Hexyl-3-methylimidazolium hexafluorophosphate
[Hmim][TfO]	1-Hexyl-3-methylimidazolium trifluoromethanesulfonate
[HNEt_3_][HSO_4_]	Triethylammonium hydrogen sulfate
[HSO_4_]^−^	Bisulfate
[i-C_5_mim]^+^	1-(Isopentyl)-3-(3-triethoxysilylpropyl)imidazolium
[i-C_5_TPIm][Cl]	1-(Isopentyl)-3-(3-triethoxysilylpropyl)imidazolium chloride
[i-C_5_TPIm][NTf_2_]	1-(Isopentyl)-3-(3-triethoxysilylpropyl)imidazolium chloride bis(trifluoromethanesulfonyl)imide
[ImNH_2_][BF_4_]	1-Aminoimidazolium tetrafluoroborate
[IPDAH][Im]	Isophorone diamine imidazolate
[MeTBDH]_2_[HFPDO]	1-Methyl-1,5,7-triazabicyclo[4.4.0]dec-5-enium perfluorinated diol-derived dianion
[methide]^−^	Tris(trifluoromethylsulfonyl)methide
[MTBD][Im]	7-Methyl-1,5,7-triazabicyclo[4.4.0]dec-5-ene imidazolate
[MTBDH][TFE]	7-Methyl-1,5,7-triazabicyclo[4.4.0]dec-5-enium 2,2,2-trifluoroethoxide
[NO_3_]^−^	Nitrate
[NTf_2_]^−^	Bis(trifluoromethanesulfonyl)imide
[OHC_3_mim][NO_3_]	1-(3-Hydroxypropyl)-3-methylimidazolium nitrate
[Omim][TfO]	1-Octyl-3-methylimidazolium trifluoromethanesulfonate
[P_4442_]_2_[IDA]	Tri-n-butylphosphonium iminodiacetate
[P_66614_][2-Op]	Trihexyl(tetradecyl)phosphonium 2-oxypyridinate
[P_66614_][3-HMPz]	Trihexyl(tetradecyl)phosphonium 3-hydroxymethylpyrazolate
[P_66614_][4-ABI]	Trihexyl(tetradecyl)phosphonium 4-aminobenzimidazolate
[P_66614_][4-Me-PhO]	Trihexyl(tetradecyl)phosphonium 4-methylphenoxide
[P_66614_][4NH_2_-NC]	Trihexyl(tetradecyl)phosphonium 4-aminonicotinate
[P_66614_][Im]	Trihexyl(tetradecyl)phosphonium imidazolate
[P_66614_][Ind]	Trihexyl(tetradecyl)phosphonium indolide
[P_66614_][p-AA]	Trihexyl(tetradecyl)phosphonium p-aminobenzoate
[P_66614_][PPhO]	Trihexyl(tetradecyl)phosphonium phenylphosphonate
[P_66614_][Triz]	Trihexyl(tetradecyl)phosphonium triazolate
[P_66614_]^+^	trihexyl(tetradecyl)phosphonium
[P_8883_][NTf_2_]	Trihexyltetradecylphosphonium bis(trifluoromethylsulfonyl)imide
[PF_6_]^−^	Hexafluorophosphate
[Pmim][NTf_2_]	1-Propyl-3-methylimidazolium bis(trifluoromethylsulfonyl)imide
[S_222_][NTf_2_]	Triethylsulfonium bis(trifluoromethylsulfonyl)imide
[SCN]^−^	Thiocyanate
[TfO]^−^	Trifluoromethanesulfonate
[TMA][HPy]	Tetramethylammonium 2-hydroxypyridinate
